# Detection and validation of common noctule bats (*Nyctalus noctula*) with a pulse radar and acoustic monitoring in the proximity of an onshore wind turbine

**DOI:** 10.1371/journal.pone.0299153

**Published:** 2024-06-12

**Authors:** Polina Krapivnitckaia, Jannes Kreutzfeldt, Helge Schritt, Holger Reimers, Carolin Floeter, Michael Reich, Veit Dominik Kunz

**Affiliations:** 1 Competence Center for Renewable Energies and Energy Efficiency (CC4E), Hamburg University of Applied Sciences, Hamburg, Germany; 2 Department of Environmental Technology, Faculty of Life Sciences, Hamburg University of Applied Sciences, Hamburg, Germany; 3 Büro für Umweltkartierung—Informationsverarbeitung—Naturbewertung (U-I-N), Pinneberg, Germany; 4 Institute of Environmental Planning, Leibniz University Hannover, Hannover, Germany; 5 Department of Process Engineering, Faculty of Life Sciences, Hamburg University of Applied Sciences, Hamburg, Germany; National Institute of Technology Srinagar, INDIA

## Abstract

This paper presents the results of bats detected with marine radar and their validation with acoustic detectors in the vicinity of a wind turbine with a hub height of 120 m. Bat detectors are widely used by researchers, even though the common acoustic detectors can cover only a relatively small volume. In contrast, radar technology can overcome this shortcoming by offering a large detection volume, fully covering the rotor-swept areas of modern wind turbines. Our study focused on the common noctule bats (*Nyctalus noctula*). The measurement setup consisted of a portable X-band pulse radar with a modified radar antenna, a clutter shielding fence, and an acoustic bat detector installed in the wind turbine’s nacelle. The radar’s detection range was evaluated using an analytical simulation model. We developed a methodology based on a strict set of criteria for selecting suitable radar data, acoustic data and identified bat tracks. By applying this methodology, the study data was limited to time intervals with an average duration of 48 s, which is equal to approximately 20 radar images. For these time intervals, 323 bat tracks were identified. The most common bat speed was extracted to be between 9 and 10 m/s, matching the values found in the literature. Of the 323 identified bat tracks passed within 80 m of the acoustic detector, 32% had the potential to be associated with bat calls due to their timing, directionality, and distance to the acoustic bat detector. The remaining 68% passed within the studied radar detection volume but out of the detection volume of the acoustic bat detector. A comparison of recorded radar echoes with the expected simulated values indicated that the in-flight radar cross-section of recorded common noctule bats was mostly between 1.0 and 5.0 cm^2^, which is consistent with the values found in the literature for similar sized wildlife.

## Introduction

Bats play an important role in our ecosystem and are at least partially protected in many parts of the world. Whilst in the US only several species are protected at the federal level under the Endangered Species Act, with the individual state laws extending the protection, all bat species within the European Union are under strict protection under Annex IV of the Habitats Directive (Council Directive 92/43/EEC).

Many bat species are vulnerable to operating wind turbines [1, 2]. The Global Wind Energy Council forecasts a compound annual growth rate of 15% in installed capacity for wind power between 2023 and 2027 globally, with a 12% growth rate for onshore capacity [3]. In addition, rotor diameter and hub height of new wind turbines have been continuously increasing over the years [4]. This trend raises the question of whether conventional non-intrusive acoustic detection methods can provide enough useful data as wind turbines increase in size and number.

Detecting bats has always been challenging since many species are typically nocturnal, small, and fast flyers. Acoustic monitoring allows the identification of many echolocating bat species worldwide [5–14]. However, this is challenging due to overlapping frequencies amongst different species and variations of bat calls caused by changes in the environment [15–17]. Additionally, acoustic monitoring studies can only provide information on the species’ presence and relative abundance but cannot identify the number of individuals. The resulting activity metrics are, therefore, rather abstract. Depending on the selected methodology, a commonly used metric known as "bat pass" can be defined differently, e.g., as a single echolocation call or as several echolocation calls with less than 1 s between each call [18], as a single echolocation call or as several echolocation calls within a 5 s interval [19], or as a 2 – 20 s recording with several bat calls [20], to name just a few. Thus, comparing the results of different acoustic monitoring studies can be challenging. Furthermore, the detection range of acoustic detectors is species-dependent. Compared to wind turbines (WTs) with a blade length of 60 m, an acoustic detector placed at the bottom of the nacelle can only cover 23% of the rotor-swept area for bat calls at 20 kHz (the fundamental frequency of *Nyctalus noctula*) and approximately 4% for calls at 40 kHz (the fundamental frequencies of, e.g., *Pipistrellus kuhlii* and *Pipistrellus nathusii*) [21]. Installing an additional microphone on a WT’s tower can certainly help to increase the bat detection capabilities at or near the rotor-swept area and improve the assessment of bat activity at the WT [21, 22]. However, such microphones are stationary and cannot follow a nacelle’s rotational movement. Despite the limited detection area, Behr et al. [23] argue that standardized and referenced acoustic monitoring at WTs’ nacelles can be used for the reliable estimation of bat fatalities as well as for determining curtailment algorithms for the bat-friendly operation of wind turbines.

Radar technology has been widely used for bird detection [24–35], but rarely considered for bats [36–38]. Flocks of Brazilian free-tailed bats (*Tadarida brasiliensis*) were tracked with weather surveillance radars and analyzed with respect to their relative colony size and their general spatial and temporal movement patterns [39–42]. However, weather surveillance radars cannot provide high-resolution data or information on individuals [43]. Dedicated tracking radars can be used to study the flight paths of individuals. Since tracking radars focus on single targets, they lack broadscale surveillance capabilities and have therefore not been frequently used for bat studies [43]. Two previously captured and categorized bat species, a common noctule bat (*Nyctalus noctula*) and a serotine bat (*Eptesicus serotinus*), were successfully tracked with a military tracking radar over a distance of approximately 600 m [44]. Kunz et al. [43] mention that marine radars allow monitoring of individual bats and can, depending on the antenna position, measure flight altitude or gather horizontal flight information. Using the combination of a marine radar with a parabolic antenna in a vertical setup and an infrared camera system, Gauthreau et al. [31] detected bats at heights up to 1343 m. Werber et al. [45, 46] employed a vertical X-band radar with a rotating horn antenna to detect bats at altitudes of up to 800 m. Lastly, a report by Ahlén et al. [47] indicated that *N*. *noctula* had been successfully tracked with a radar, but the authors did not specify which equipment they used in their study.

Most of the published studies on radar bat detection lack a systematic assessment of detection probability within the surveyed volume of the radar. The detection probability is strongly dependent on the radar specifications as well as the radar cross-section (RCS) of the species to be detected. Mirkovic et al. [48] found that the mean RCS of *T*. *brasiliensis* measured with an X-band radar was 1.49 – 2.78 cm^2^, depending on the angle of incidence of the radar beam. However, the authors did not provide estimations of the detection range of their equipment. With the help of an artificial bat and an X-band radar, Kreutzfeldt et al. [49] concluded that *N*. *noctula* has an RCS of 12.7 cm^2^ when fully exposed to the antenna beam. The rather conservative threshold of the intensity was 25 out of 32 levels and the detection range was up to 800 m in the main beam of the slotted waveguide antenna. Werber et al. [46] found the detection range to be up to 800 m for bats like the *Pipistrellus pipistrellus* for a vertical X-band radar BirdScan MR1, with its manufacturer stating the range up to 1000 m for bats in general. Again, no detection or visibility criteria were mentioned for these estimations.

Nicholls and Racey [50, 51] studied the influence of electromagnetic radiation from several radars on the activity of several bat species. When exposed to electromagnetic fields greater than 2 V/m, bat activity was significantly reduced within 200 m of weather radars and civil and military air traffic control radar stations. However, with no access to the restricted information on the radars, the authors could not evaluate the influence further [50]. A follow-up study with a portable X-band pulse radar (6 kW peak power) revealed that bat activity was reduced within 30 m of the non-rotating slotted waveguide antenna, but no significant reduction was noticed for a configuration with a rotating antenna [51]. A study by Gilmour et al. [52] with a portable X-band pulse radar (6 kW peak power) who analyzed the bat activity within a 30 m radius of the antenna concluded that electromagnetic radiation was an ineffective deterrent for bats. Thus, it can be concluded that a portable, low-output power marine X-band radar could be used to capture typical, non-altered bat activity, especially at considerable distances from the antenna.

One of the main challenges when using radar for biological studies is an objective and accurate differentiation between targets, e.g., birds and bats. A speed threshold of 6 m/s can be used as a separator of most insects from birds and bats. However, separating bats from birds by speed alone does not seem to be possible [43]. Werber et al. [46] investigated bats with a BirdScan MR1 radar. The authors rely on a combination of measured altitude, speed, radar cross-section, wing-flapping frequency, average pulse and pause length to classify potential targets as bats. However, the data used to train their classification algorithm was selected by discarding the targets with non-bat-like characteristics. The algorithm was validated by achieving an intentionally low success rate when applied to a data set without any bats. In contrast to this method, other radar studies typically employ acoustic detectors or thermal cameras to prove the presence of bats in or near the studied area [e.g. 45, 53]. We have not yet encountered any published study in which bat movements recorded by radar were directly associated with acoustic detection and consequently validated as bat tracks.

In this study, we advance the work of Kreutzfeldt et al. [49] by identifying and validating bat tracks around the nacelle of a WT, with a focus on the *N*. *noctula* species. The applied combination of an acoustic bat detector (ABD) and a horizontal X-band pulse radar, allowed us to track individuals and associate the tracks with the bats. For the purposes of the data association, this study was limited to a volume in the vicinity of one WT, even though the radar setup could monitor a much larger volume. The selected WT had a 120 m hub height and 117 m rotor diameter. The WT was chosen because it allows for mounting an ABD at high altitudes whilst providing easy access. In contrast to Kreutzfeldt et al. [49], the radar was modified by inclining the antenna beam slightly upwards, towards the nacelle.

As stated earlier, radar technology is not commonly used for bat studies. However, the large detection volume of this technology motives us to further explore its potential for bat detection. The prime aim of this study was to validate the detection of common noctule bats by X-band pulse radar. This can be achieved by analyzing bat tracks extracted from the radar images near the ABD and matching them to the recorded bat calls. A further aim was to compare extracted the average RCS of the detected bats and their average speed to the values found in literature for further validation of bat tracks. A final aim was to confirm the possible radar detection range of bats based on measurements and simulations. The results developed in this study can be applicable internationally as the importance of bat detection is relevant for multiple applications across the globe, e.g. wind energy projects.

## Materials and methods

### Study site and measurement equipment

All our experiments were carried out in a wind park in Curslack in Hamburg, Northern Germany. The wind park consists of five wind turbines with rotor diameters of 117 m, hub heights of 120 m, and a cumulative output capacity of 12.6 MW. The WTs are located on flat, agricultural land with many small ditches and a few isolated trees. Other landscape structures, like woods, bushes, buildings, and a motorway, can be found in the surrounding area.

The Curslack wind park is part of the research facilities of the Hamburg University of Applied Sciences. Access and use of the wind park’s territory was granted by the university and owners of the agricultural land. Each entry to a WT and installation of measurement equipment was coordinated with the operator of the wind park. The permit to use the 9410 MHz radar frequency was obtained from the respective authorities (permit Nr 31430399 from Bundesnetzagentur für Elektrizität, Gas, Telekommunikation, Post und Eisenbahnen). All involved personnel were instructed on the safety measures regarding working in the wind park.

Seven out of 13 bat species present in the Hamburg region [54] were detected in this wind park in a survey conducted in 2015 [55]. This study focused on *Nyctalus noctula*, since they are known to have a high collision risk with WTs [56], and they are the biggest species in size (with a typical body mass between 21 and 30 g) found in the wind park.

A commercially available ABD GSM-batcorder (ecoObs GmbH, Nuremberg, Germany) was installed in a nacelle of a WT with the settings recommended for long-term nacelle monitoring: Automated recording within a defined scanning period; 500 kHz sampling rate; threshold − 36 dB, quality 20; critical frequency 16 kHz; post trigger 200 ms. According to Weber et al. [57], the mean detection range of this ABD is about 41 m for signals of 20 kHz, which matches the fundamental frequency of *N*. *noctula*. Acoustic recordings were saved on SD cards with second-precision timestamps. Due to restricted access to the nacelle, manual synchronization of its internal clock happened infrequently, during the exchange of the SD cards, leading to a clock drift of the ABD.

**Fig 1:**
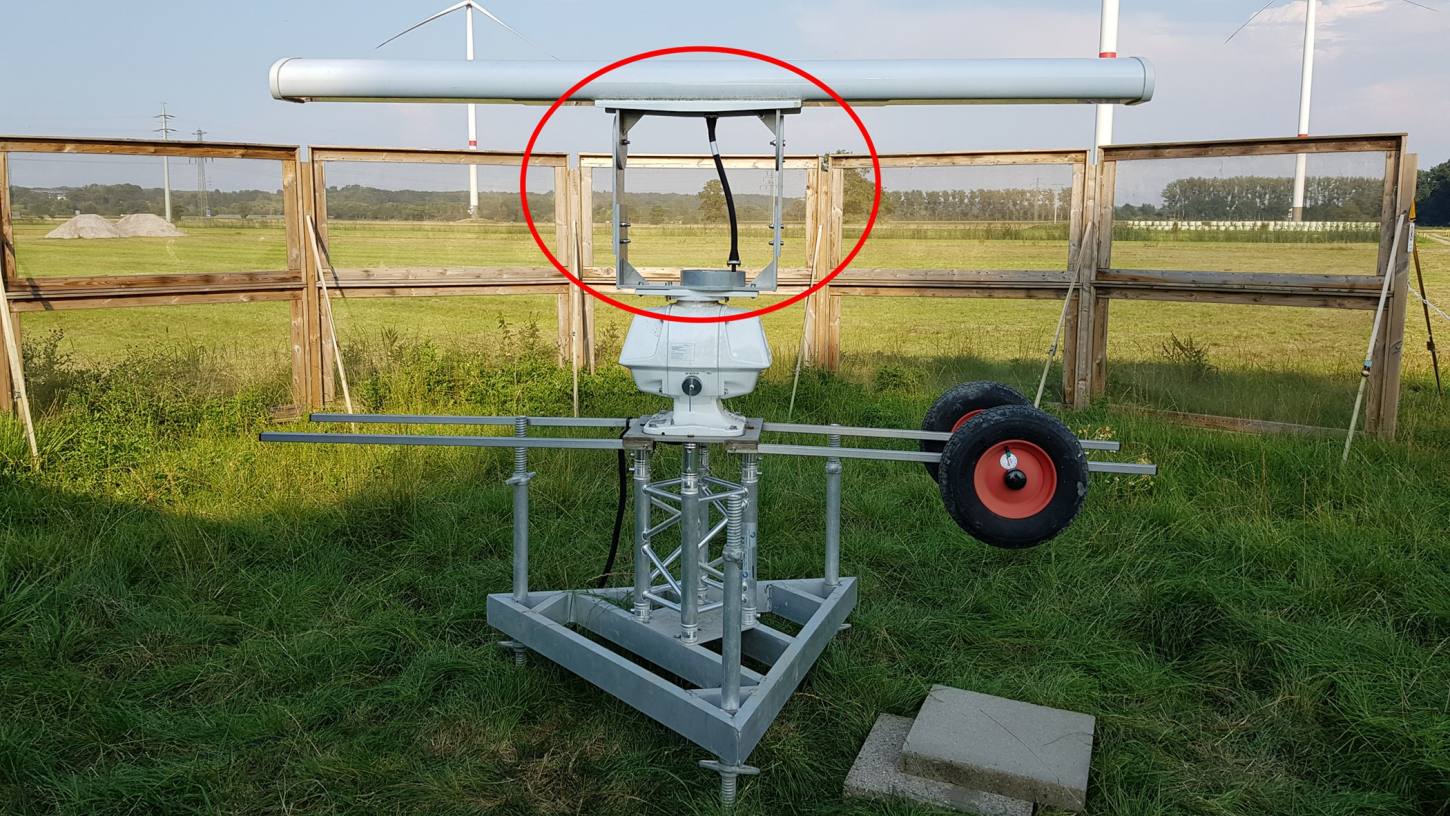
Modified FURUNO FAR 2117 marine radar setup used in this study. The red oval indicates the modified part of the radar–the waveguide in between aluminum panels for a custom adjustable vertical angle of the antenna. The clutter shielding fence is visible behind the radar, with three towers of the wind turbines in the background.

To record the bats’ movement in the wind park, we used a portable X-band pulse radar FAR 2117 (FURUNO Electric Co., Ltd., Nishinomiya, Japan; 12 kW output power) positioned approximately 665 m from the WT, where we installed the ABD at nacelle height. The approximate distance between the radar antenna and the ABD was 675 m. Kreutzfeldt et al. [49] estimated that *N*. *noctula* can be detected at a distance of up to 800 m in the main antenna beam of the FAR 2117 radar and that a clutter shielding fence with a 1 mm mesh spacing placed 6 m away from the radar can significantly reduce unwanted electromagnetic reflections (clutter) from the ground. However, in comparison to [49], we applied several modifications, mainly regarding the antenna position. The antenna mounting position was altered so that the slotted waveguide antenna could rotate in the horizontal plane with a vertical inclination of 15° to decrease clutter, as shown in Fig 1. This modification led to the extension of the waveguide using commercially available parts (a flexible waveguide and two h-bends) and two custom-made adapter plates to mount the antenna to the transceiver. The additional insertion losses of the waveguide extension summed up to 1.28 dB.

The radar processor was set to cover a range of 1.5 km, 0.07 μm pulse length, 3000 Hz pulse repetition frequency, head-up presentation mode, interference rejection setting 2 (IR 2), no echo stretch (ES OFF), no echo averaging (EAV OFF), no rain clutter suppression (AUTO RAIN OFF; A/C RAIN 0), no sea clutter suppression (SEA MAN 0), automatic tuning of the receiver (TUNE AUTO), no white noise rejection (NOISE REJ OFF), video contrast 2-B, color set BRILL1 and brilliance level of display 100.

To assess the detection capabilities of the radar setup, we further developed the analytical simulation model presented in [49]. This model simulates the expected received power as a function of distance and height from the radar and ground level, respectively. The following input parameters were used: radar transmitting power, 2D antenna gain, antenna position, the wavelength of the radar, RCS of the object, a threshold of value of the reflected power, distance to clutter shielding fence, and height of clutter shielding fence. However, to simulate the radiation profile for this study, the model was extended to account for the antenna inclination of 15° and the added losses caused by the flexible waveguide.

**Fig 2:**
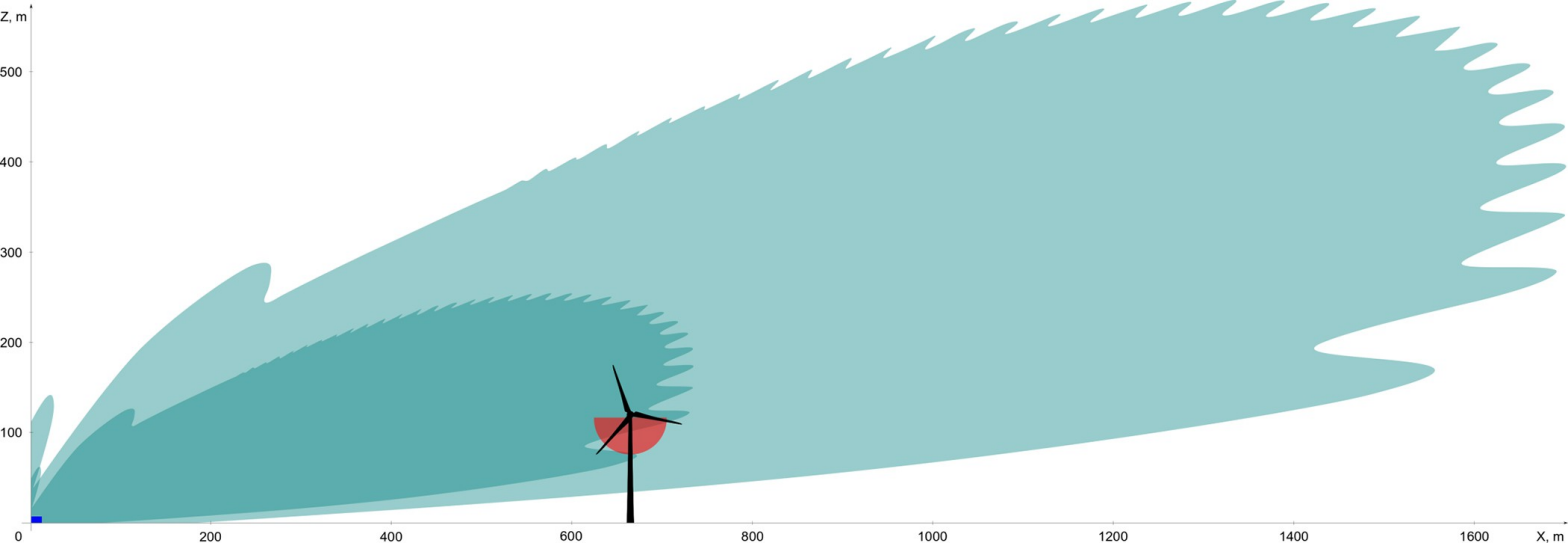
Schematical representation of the measurement setup in the wind park. The location of the radar setup (radar and clutter shielding fence) is indicated by the small blue rectangle at the origin of the coordinate system. The simulated detection volume of our marine radar (RCS = 12.7cm^2^, antenna inclination angle 15°) is in turquoise, with more saturated shade for the threshold of -74 dBm and less saturated for -89 dBm. The simplified shape of the detection volume of the ABD (for bat calls with frequencies ≤ 20 kHz) is in red.

A representation of the typical cross-section of the simulated radar detection volume for a common noctule bat with an RCS of 12.7 cm^2^, as well as the WT’s size and location in relation to the radar setup, are schematically illustrated in Fig 2. The maximum radar detection volume is found by rotation of the illustrated cross-section by 360° around the z-axis. The size of the cross-section is influenced by the selected threshold of the received power. For comparison, the simplified detection volume covered by the ABD for typical calls of a common noctule bat with a radius of 41 m at the bottom of the nacelle is presented in red.

**Fig 3:**
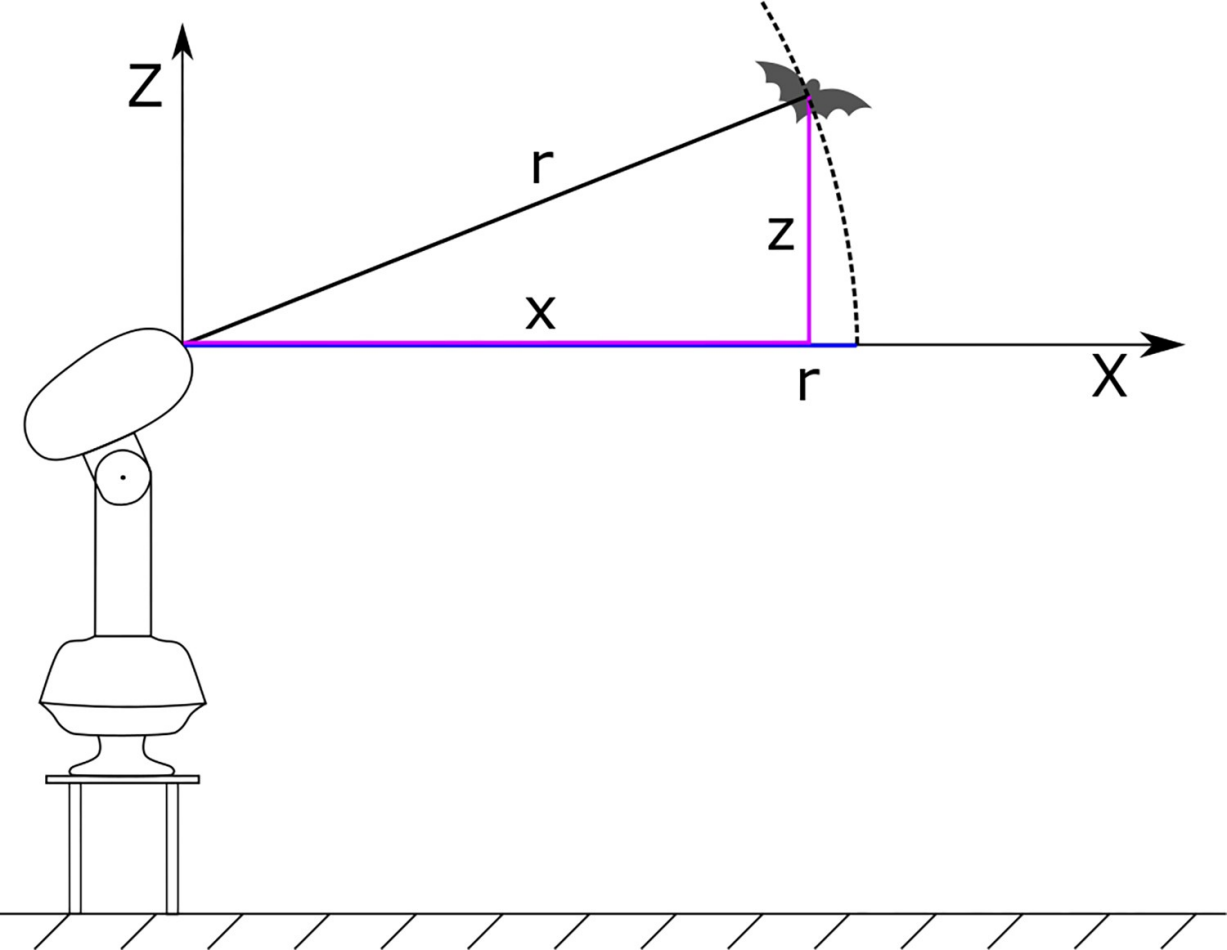
Measured distance from the radar antenna to an object. x—horizontal distance of an object to the radar antenna; z—elevation of an object over the radar antenna; r—object’s distance along the radar beam, which is traced directly on the 2D radar image.

The FAR 2117 has a plan position indicator display, which is updated at 2.5 s intervals with each antenna rotation. The resulting polar coordinates of a detected object are the corresponding antenna rotation angle and the shortest distance of the object to the antenna illustrated in Fig 3 as the distance *r*. It should be noted that although the object’s distance along the radar beam *r* can be projected into a cartesian coordinate system as a horizontal distance *x* and elevation *z*, individual information of *x* and *z* is not available. Instead, the object’s distance *r* is directly traceable on the 2D radar image. With no information about *x* and z, uncertainty about the object’s actual position is introduced since the object can be anywhere within the antenna beam on a circumference with the radius *r*.

We used a video capture device (Epiphan DVI2USB 3.0) to record the radar images at the rate of the antenna rotation speed and stored them on a server. To ensure the correct timestamp of an image, the server was synchronized daily with a network time protocol server.

### Preliminary radar measurements

Our analytical simulation model allowed us to obtain the received signal power (in dBm) for objects with defined RCSs at a known vertical and horizontal distance from the radar. In contrast, the information available from the radar images for an object’s echo consist of a number of illuminated pixels and their brightness intensity. The intensity is expressed as levels in the range of 1 to 32 (1 represents black, 32 represents maximum brightness). From [49], we know that intensity level 25 corresponds to the received signal power of -74 dBm (later referred to as the "theoretical value"). A series of field experiments was conducted to investigate the correlation between the observed intensity level and the received signal power.

**Fig 4:**
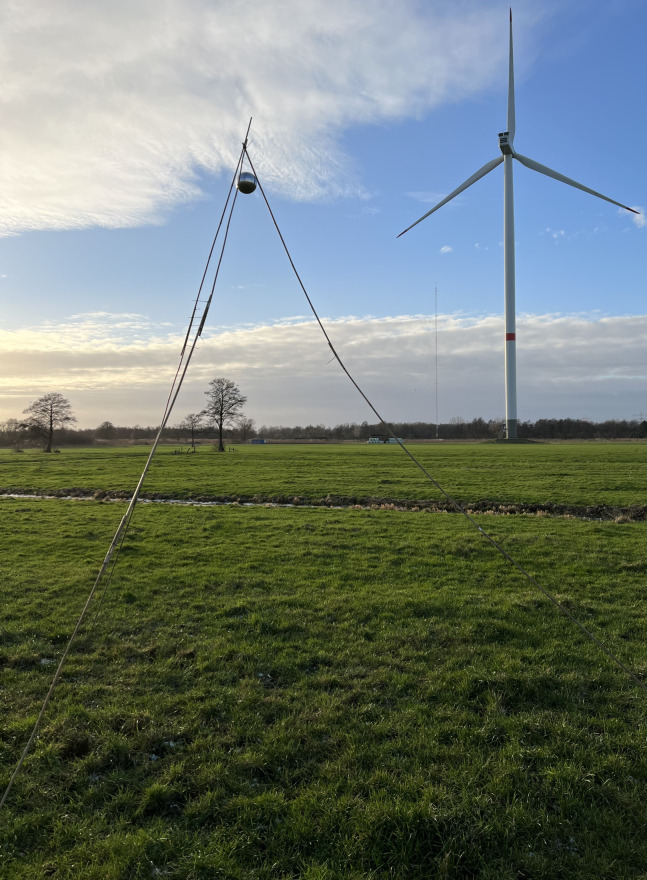
Field measurements of intensity levels of metal spheres with known RCS. Tripod, made from thin, dry bamboo rods, holding a polished hollow aluminum sphere of 150 mm diameter.

Ideally, the preliminary experiments are executed with a perfectly conducting sphere since it is easy to calculate its RCS [58]. In our experiments, we used polished hollow aluminum spheres of 40, 80, 100, and 150 mm diameters, with corresponding ideal RCSs of 11.3, 42.6, 80.8, and 172 cm^2^, respectively. We suspended the spheres at a height of 4–4.5 m above ground using a tripod made from thin, dry bamboo rods (see Fig 4). In the context of the shape of the radar detection volume (see Fig 2), the measurement locations were near the lower edge of the simulated detection volume. A total of 20 measurements were made at five locations with minimal ground clutter and three different antenna heights for approximately 140 s (approximately 55 images) per measurement. Since the field experiments required a re-assembly of the tripod for each new measured object and measurement location, we estimated the uncertainty of the vertical position of a measured object in the field to be within ±0.5 m. The horizontal distance of the measured object from the radar was calculated from the radar images, and the uncertainty of the horizontal position was estimated to be within ±1 pixel or ±3 m.

The intensity levels were calculated for each radar image according to [49] as the mean value of the intensities of a 3 x 3 pixel matrix around the brightest pixel of the radar echo. Since several images were taken per measurement, the resulting median intensity level per measurement sample was used for further analysis. The corresponding values of simulated received signal power were extracted from our modified analytical simulation model. Regression analysis was applied to find the dependence between the simulated received signal power and the measured intensity level. For the regression analysis, median intensity levels higher than level 1 were considered the dependent variable, and the respective simulated values of the received power were the independent variable. Since the assumption of homogeneity appeared to be violated, the Bisquare weighted robust regression method was applied.

Prior to the measurements, the radar was calibrated according to the procedure proposed in [49] to ensure measurements were comparable. This procedure compensates for the gradual degradation of the magnetron and, consequently the received signal, by adjusting the sensitivity of the receiver.

In addition, four measurements were conducted to test the influence of the deliberately raised sensitivity of the receiver on the intensity level of an object. These measurements were conducted at a single location with 2 test objects, with the gain knob raised by 1–2 positions from the calibrated position.

### Data selection and evaluation

The data for this study was recorded in 2020. While the ABD was employed for nacelle monitoring throughout the whole season, from mid-March to mid-November, the radar was in operation cumulatively for nearly 21 weeks. For this data analysis, we only focused on the data recorded during time intervals that met the following criteria:

- Both acoustical and radar recordings were in operation.
- After sunset and before sunrise.
- No precipitation.
- The ambient temperature measured at WT’s nacelle height was greater than one established in the blanket curtailment of the WT (*T*_*amb*_ *> 6*°*C*).

Although it is often recommended that bat monitoring should begin shortly before sunset and end shortly after sunrise, for this data analysis, we avoided adding these additional time intervals, to exclude the possible presence of diurnal birds.

The general workflow of our data analysis comprised the following steps:

1. Acoustic data evaluation.
2. Selection of "bat events" with common noctule bat calls.
3. Manual identification of tracks on radar images during the selected bat events.
4. Identification of tracks with the potential to be aligned with recorded bat calls.
5. Temporal alignment of acoustic data to the radar timeline.

Since regular clock synchronization of the ABD was not feasible, clock drift and, thus, drift in the timestamps of recordings were inevitable. In preparation for the temporal alignment of acoustic data to the radar’s timeline, we calculated the potential worst-case scenario of the clock drift. The longest period between the manual clock synchronization and the furthest unsynchronized recording session was approximately 188 days. Although [59] measured larger values of drift for hand-held recording devices than the typical specified accuracy of ± 1 ppm (± 1 μs/s) for devices with crystal oscillators, the clock drift values that we observed for our bat detectors throughout the long-term measurements in the field were predominantly within ± 0.4 ppm. For the potential worst-case estimation, the conservative value of ± 1 ppm was selected, resulting in a maximum error of ± 16 s over the longest unsynchronized period. Thus, the actual timestamps of acoustic recordings were estimated to be within a time slot of *Δt*_*unsync*_ *= ± 16 s* from the recorded timestamp.

The acoustic data was evaluated using automatic evaluation software bcAdmin 4 (ecoObs GmbH, Nuremberg, Germany), followed by manual control of two independent experts according to [11] with the focus on *Nyctalus noctula* species. We quantified acoustic bat activity using the following metrics: number and duration of recordings, number and duration of sequences, and duration of active time. We defined a sequence as a series of consecutive recordings with bat calls of the same group with a time interval of no more than 2 s from each other. The assumption behind this is that not many individuals would be present at nacelle height simultaneously, meaning different sequences would likely belong to different individuals. We defined the term active time as the time interval containing one or several adjacent sequences, counted from the start of the first sequence to the end of the last sequence within a bat event.

**Fig 5:**
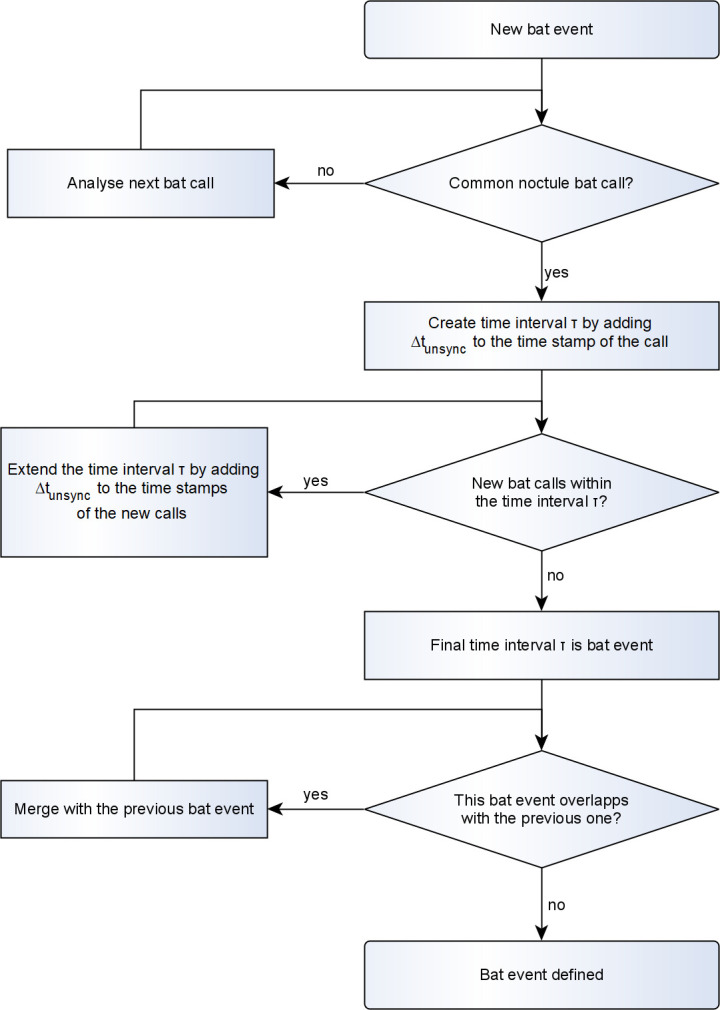
Procedure to identify a bat event.

Once the characteristic calls of common noctule bats were identified, time intervals termed "bat events" were created (Fig 5). The resulting bat events contained at least one call of a noctule bat and had an estimated clock drift time slot of *Δt*_*unsync*_ *= ± 16 s* added to and subtracted from the timestamps of the corresponding outer recordings. The bat events were then used to select radar images for bat tracking.

Once the radar images corresponding to the bat events were selected, the manual identification of tracks commenced. To keep the process unbiased, the information on the timing and length of bat call sequences was not accessed during the track identification on the selected radar images.

**Fig 6:**
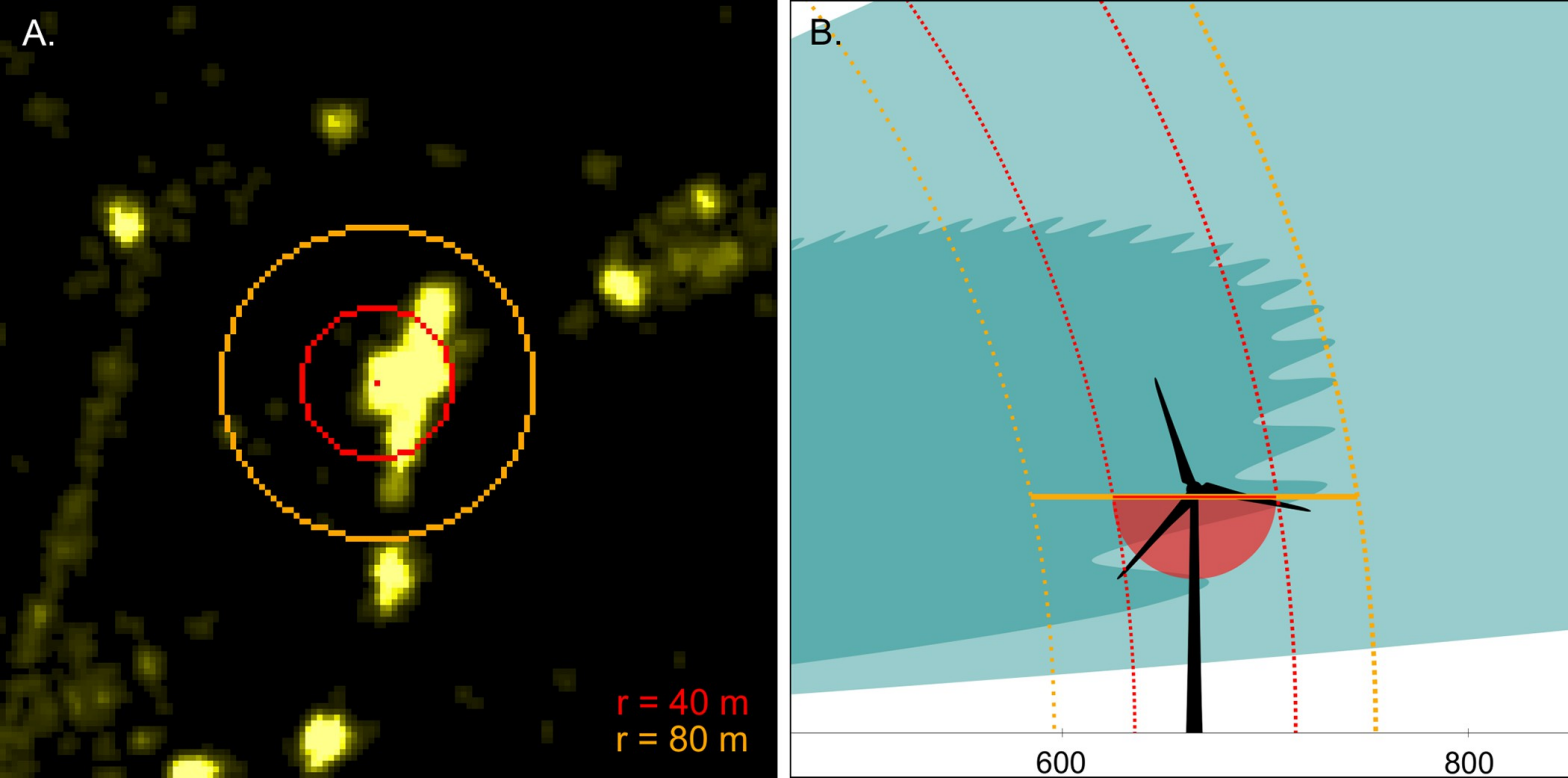
Two areas of interest around the microphone, with radii of 40 and 80 m. (A) Location of the areas of interest on the radar image, (B) vertical projection of boundaries of the areas of interest.

For this study, we limited the radar investigation area to approximately 400 x 400 m, corresponding to a 135 x 135 pixel image with the WT being in the center of the image (Fig 6A). In addition, we only considered tracks with at least one radar echo within the circumference with the radius r = 80 m (the orange circumference in Fig 6A) centered at the location of the ABD. Although this circumference is larger than the one representing the detection volume of the ABD (r = 40 m, for signals of frequency 20 kHz, which match typical calls of *N*. *noctula*, red circumference in Fig 6A), the outer circumference was selected to include tracks otherwise not visible within the acoustic detection radius due to high intensity level of the WT’s radar echo. It should be noted that since only the object’s distance in the radar beam is available from the radar images, the objects crossing the circumferences could be anywhere within the corresponding vertical projections of the circumferences in the antenna beam (Fig 6B).

Additionally, we developed several criteria for the movement of radar echoes to be qualified as a track:

- A track must consist of at least three consecutive radar echoes which gradually change their position from image to image and are within approximately 20 pixels from each other.
- The average projected track speed cannot be higher than 19.4 m/s given in [60] for a noctule bat; should it be above this value, the track would be excluded from further evaluation.
- For each of the three required consecutive radar echoes, the intensity level of a 3 x 3 pixel matrix around the brightest pixel of a radar echo must be higher than intensity level 1 (black); any further radar echoes of the track, must be at least 1 pixel in size and brighter than the intensity level 1.
- All radar echoes of a track must appear on consecutive radar images—no discontinuity is allowed.

The constraints of our track definition listed above were developed to ensure reproducibility and to prevent flickering clutter from being considered as part of a track. Once the movement of radar echoes were qualified as tracks, we tested each track regarding its potential to be associated with corresponding bat calls. This task involved the temporal alignment of the acoustic recording with the corresponding radar timeline. To be associated with bat calls, a track was required to satisfy the following two conditions:

1. The direction of the track must either point towards or away from the circumference with r = 40 m.
2. The timing of the possible crossing of the circumference (r = 40 m) must correspond to the timing of a bat call.

Since the timestamp drift was unknown, we followed a three step process. First, we identified the track with trajectories to potentially cross the circumference with r = 40 m. A distance over time diagram was generated for each bat event, where the distance is measured between the radar echo and the ABD. The distance over time diagrams and radar images allow preselection of the relevant tracks. Second, we added the timing information of the bat calls to the distance over time diagrams. The bat calls were plotted at the maximum detection range (r = 40 m) of the ABD. Third, we aligned the acoustic data to the radar’s timeline to match with the preselected tracks. The most frequent and coherent time shift values were assumed to be the actual time shift. After the final alignment, the preselected tracks were tested again for their potential match to the previously adjusted timing of bat calls. As a result, the most likely tracks with the potential to be associated with the acoustic recording were found.

### RCS estimation of the common noctule bat

The intensity level of a pixel is dependent on the altitude, the horizontal distance from the radar antenna, the received signal power and of the object’s RCS. To estimate the RCS, we limited the dataset to tracks with the potential to be associated with bat calls. Furthermore, we only chose radar echoes which were in the r = 40 m proximity of the ABD. The intensity levels of radar echoes were calculated based on a 3 x 3 pixels matrix around the brightest pixel of the radar echo, as described in the section "Preliminary radar measurements". The intensity levels were then plotted against their distance to the radar. Comparing the extracted radar echoes with the simulation led to an approximation of the RCS.

## Results and discussion

### Preliminary radar measurements

The initial measurements were performed to determine the intensity level of radar echoes as a function of received signal power from the simulations based on a series of field experiments. The resulting median intensity levels, along with other descriptive statistics of each measurement sample, the simulated received signal power, and the measurement setup characteristics are listed in S1 Table. Rows 1–20 refer to measurements with the calibrated radar setup. Rows 21–24 refer to the additional measurements with the gain knob raised by 1–2 positions from the optimal value. Since we conducted the measurements in a field setting, external conditions such as wind gusts, moisture evaporation from the ground, as well as interference of scattered electromagnetic waves reflected from objects in the wind park might have led to some uncertainty of our measurements. Additionally, possible scratches on the surface of the metal spheres and the potential contamination of dust and dirt on the spheres’ surfaces could have also influenced the measurements.

**Fig 7:**
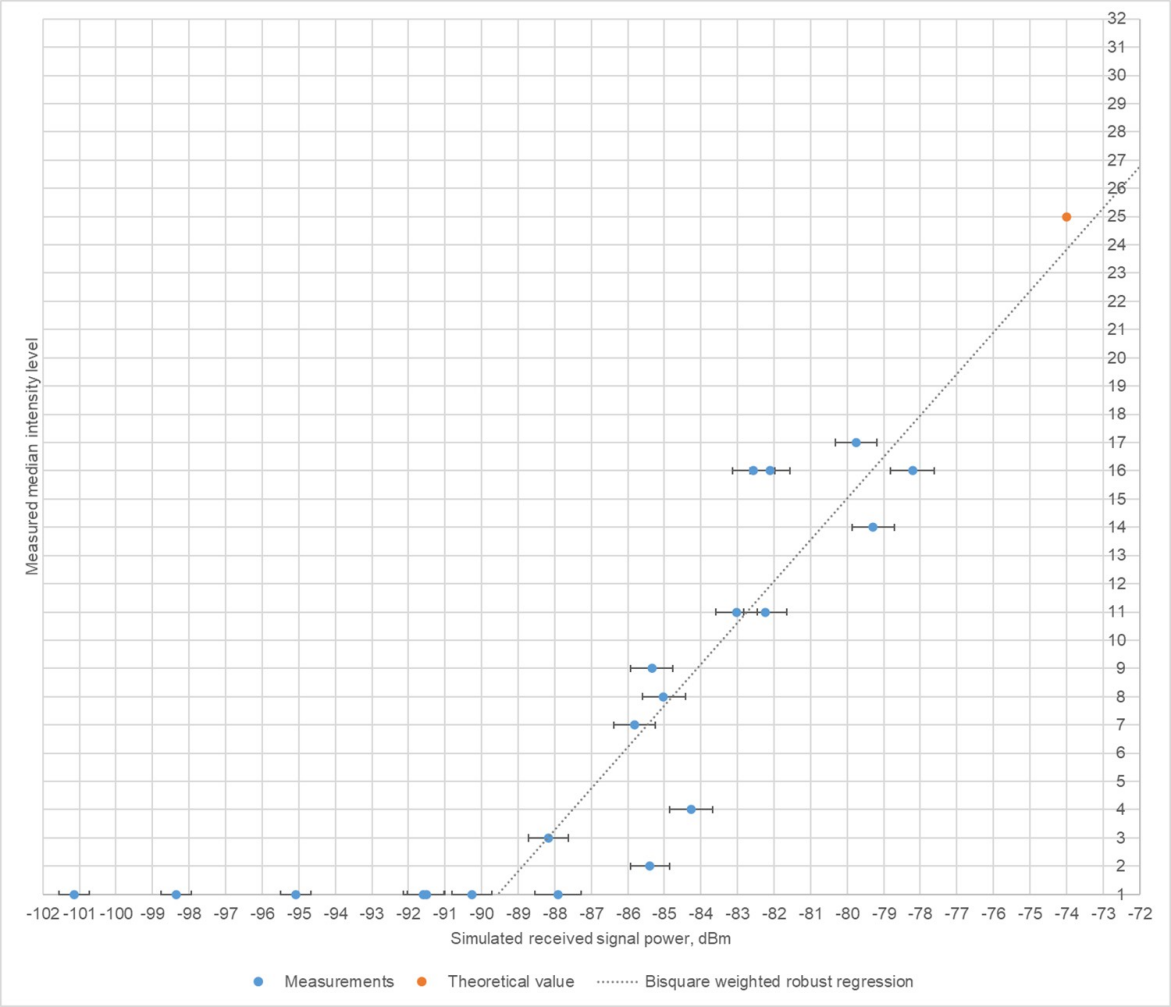
Calculated regression of measured intensity level over the simulated received power. Grey dotted line illustrates the robust regression line ***y* = 1.4666∙*x*+132.3656**. Horizontal whiskers are the range of the simulated values considering the inaccuracy of the measurement position. The orange dot is the theoretical value taken from [49] for comparison.

After applying the Bisquare weighted robust regression to the dataset from the calibrated measurements with resulting median intensity higher than level 1, we found the simulated received signal power to be positively associated with the measured median intensity (b = 1.4666, 95% CI: 0.8015, 2.1318; p = 0.0009635). The resulting regression line passes the theoretical value within 1 dBm on the scatter plot (Fig 7), confirming the validity of the calibration procedures performed before the measurements and the comparability of these measurements to those with the unmodified radar setup.

Four additional measurements were performed to determine the influence of the deliberately raised sensitivity of the receiver (gain) on the intensity level of a measured object. These measurements (results in rows 21 – 24 of Table in S1 Table) were conducted with the gain knob raised by 1 or 2 positions from the calibrated position at the same location and using the same objects as for the measurements with the calibrated gain position (results in rows 19 – 20 of Table in S1 Table). The measurements with the sphere of 100 mm diameter showed an increase in the median intensity level of 1 level per raised gain position (rows 19, 21 and 23). The measurements with the sphere of 80 mm diameter and non-calibrated gain positions (rows 20 and 22) showed the same intensity increase. However, the median intensity level from the measurement with the sphere of 80 mm diameter and with the calibrated setup (row 20) was 4 intensity levels lower than the one from the measurement with the gain position increased by 1 (row 22). Additionally, the measurement result with the calibrated gain position of the 80 mm sphere is close to the regression line (see Fig 7), while the equivalent measurement of the 100 mm sphere is further away. To facilitate the field work, we first took consecutive measurements for one object using different gain settings, before switching to another. Considering the consistency of the change in the median intensity by 1 intensity level per gain position of the five measurements and the proximity of the other measurement to the regression line, it is possible that external conditions interfered abruptly during the field measurements and influenced the results. The consistent rate of increase of 1 intensity level per gain position suggests that using a slightly suboptimal gain knob position during measurements should not alter the results dramatically.

To conclude, we estimate the uncertainty for the actual received signal power to be within ± 2 dBm from the simulated received power. Therefore, predicted intensity levels should be read from the scatter plot with a corresponding estimated uncertainty of ± 3 in intensity levels.

### Acoustic data

Of the total 5203 bat call recordings captured during the 2020 measurement season, from mid-March to mid-November, about 33% included bat calls of the group Pipistrelloid (including species *P*. *nathusii*, *P*. *pipistrellus* and *P*. *pygmaeus*), and about 67%—of the group Nyctaloid (including species *E*. *serotinus*, *N*. *leisleri*, *N*. *noctula*, *V*. *murinus*). About 48% of all recordings were associated with the *N*. *noctula* species.

**Fig 8:**
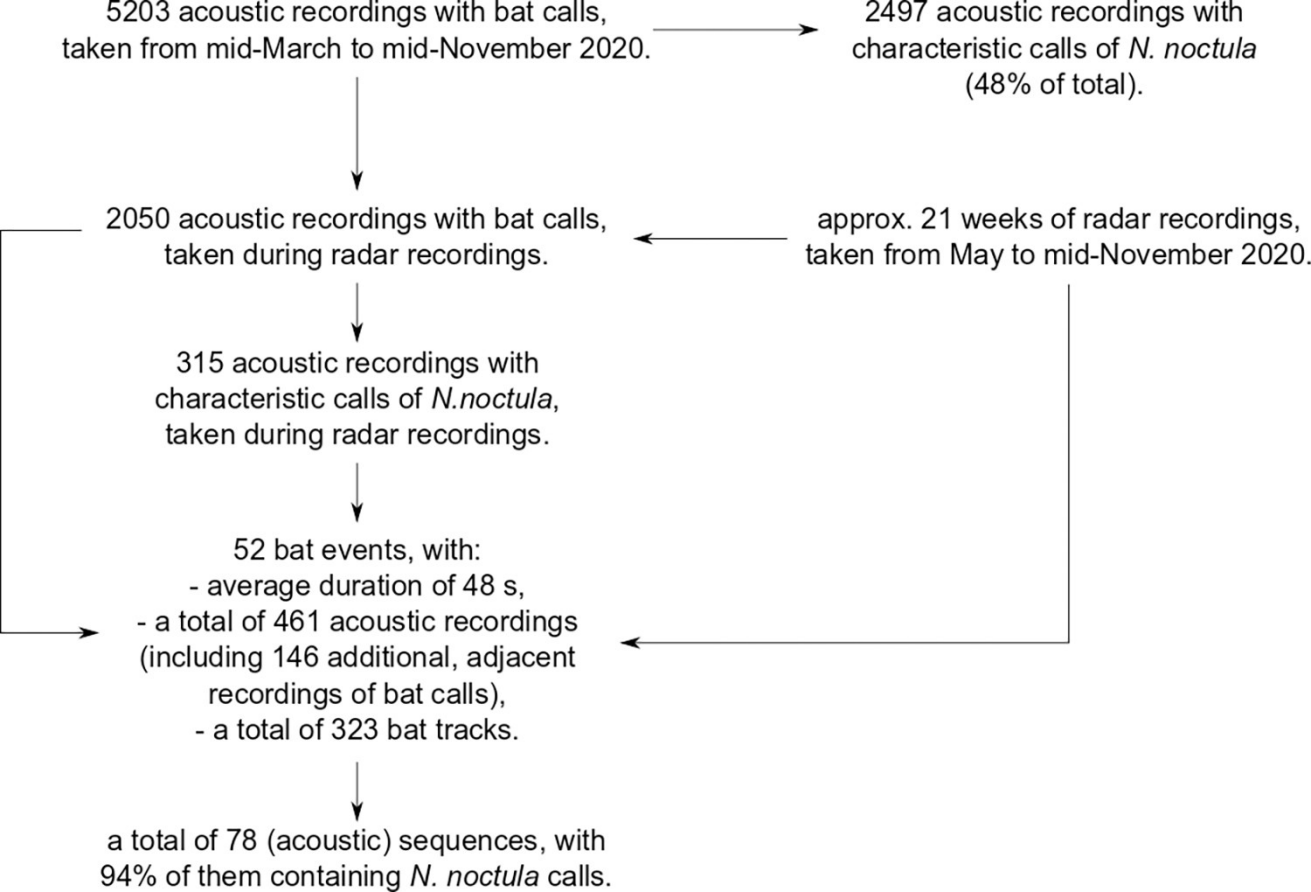
Study data from the measurement season 2020.

For this study, we analyzed data during approximately 21 weeks when both acoustic and radar monitoring were employed. The relation between the data used in this study and the data from the 2020 measurement season is illustrated in Fig 8. The temporal restriction on the data selection resulted in 2050 acoustic recordings of bat calls, of which 315 contained characteristic calls of the *N*. *noctula* species. Applying the bat event criteria from the section "Selection and evaluation of data" resulted in 52 bat events, consisting of 315 recordings with *N*. *noctula* calls (68%) and 146 additional adjacent recordings of bat calls. Detailed information on the used acoustic recordings is included in S2 Table. The mean number of acoustic recordings per bat event was 8.8 (minimum 1, maximum 41). Two bat events contained recordings with feeding buzzes of the Nyctaloid group. On average, a bat event contained 1.5 sequences (see "Data selection and evaluation" for definition), and a typical sequence lasted about 4 s (minimum 1 s, maximum 26 s). Out of the identified 78 sequences, 73 (94%) contained characteristic calls of noctule bats. The mean active time (see "Data selection and evaluation" for the definition) per bat event was 9.8 s (minimum 1 s, maximum 42 s).

### Bat events and data association

Application of the developed step-by-step procedures depicted in Fig 5 resulted in 52 bat events with a mean duration of 48 s (minimum 36 s, maximum 79 s) or approximately 20 radar images. Detailed description of the resulting bat events, including the used acoustic recordings, the radar images per each bat event, and the corresponding ambient conditions, are included in S2 Table. Ambient conditions during the bat events were predominantly favorable for bats, with a mean wind speed of 4 m/s (minimum 0.9 m/s, maximum 8.0 m/s), measured in 10 minute intervals. During seven bat events (13%), the measured wind speed was above the cut-in wind speed (6 m/s) established in the blanket curtailment for bat mortality reduction. The wind turbine was either off or slowly idling during these bat events.

**Fig 9:**
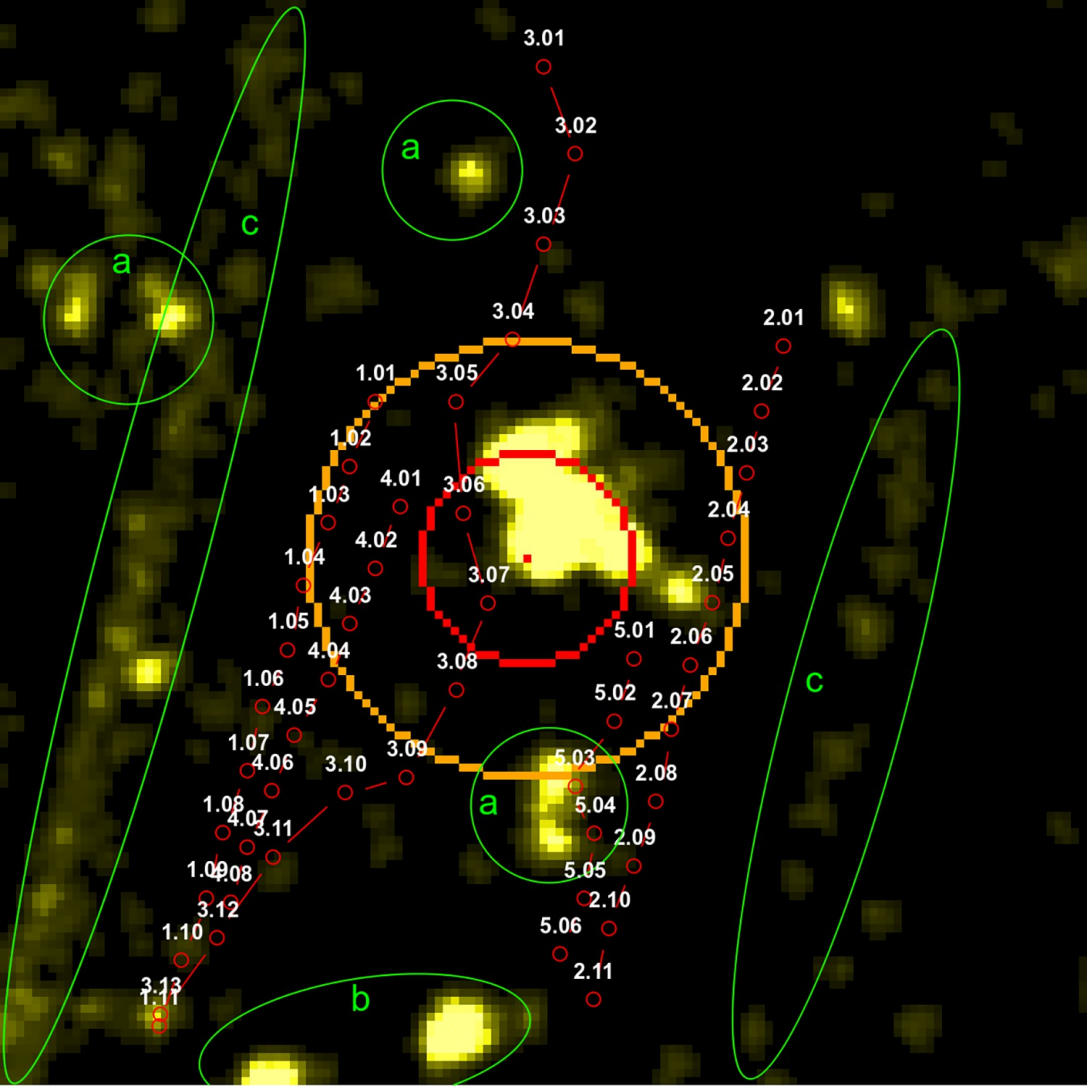
Radar image of a typical bat event with detected tracks. The large radar echo in the center of the image is the wind turbine. The red dot on the radar echo is the estimated instantaneous location of the ABD in the nacelle. The red circumference indicates the 40 m detection range of the ABD for signals with frequency f ≤ 20 kHz. The orange circumference indicates the 80 m range from the ABD. The detected tracks are plotted in red with their pathway indicated by the increasing order of enumeration. Identified clutter on the image: a—trees, b–transmission towers, c–vegetation alongside long ditches.

We identified a total of 323 tracks, which met the criteria for track selection. A bat event contained a mean of 6.2 tracks (minimum 0, maximum 19). A typical bat event is shown in Fig 9, where all tracks are plotted simultaneously on one image and the direction of a track is indicated by the increasing order of enumeration within the track (for other bat events, see S1 File). During two events (4%), no tracks were identified despite the favorable conditions for bats (wind speed between 2.7 m/s and 3.9 m/s). A possible explanation for the absence of the tracks despite the availability of bat calls could lie in the strict criteria for a track definition since several radar echo movements were identified during these two bat events.

It was possible to associate the tracking data from 43 bat events (83%) with acoustic recordings. However, not all bat call sequences (a subset of a bat event) could be associated with tracks. This effect can be explained as follows: a) the radar images did not provide tracking data which fulfilled the strict criteria for a track definition; or b) a bat whose calls were recorded by the ABD spent some time before or after the recorded call sequence in close proximity to the WT, but outside the acoustic detection volume. The proximity to the WT could have temporarily shaded its radar echo. In the latter scenario, the bat could have been tracked approaching or leaving the WT, but the extrapolation of its possible pace and trajectory towards the ABD did not temporally align with the acoustic sequence.

If several tracks aligned with a bat call sequence, all of them were marked as having the potential to be associated with the sequence since it was not possible to select specific tracks for the association. However, this scenario occurred rarely, during bat events with very high bat activity. Thus, we conclude that the majority of tracks marked as having the potential to be associated with bat calls was correctly identified.

**Fig 10:**
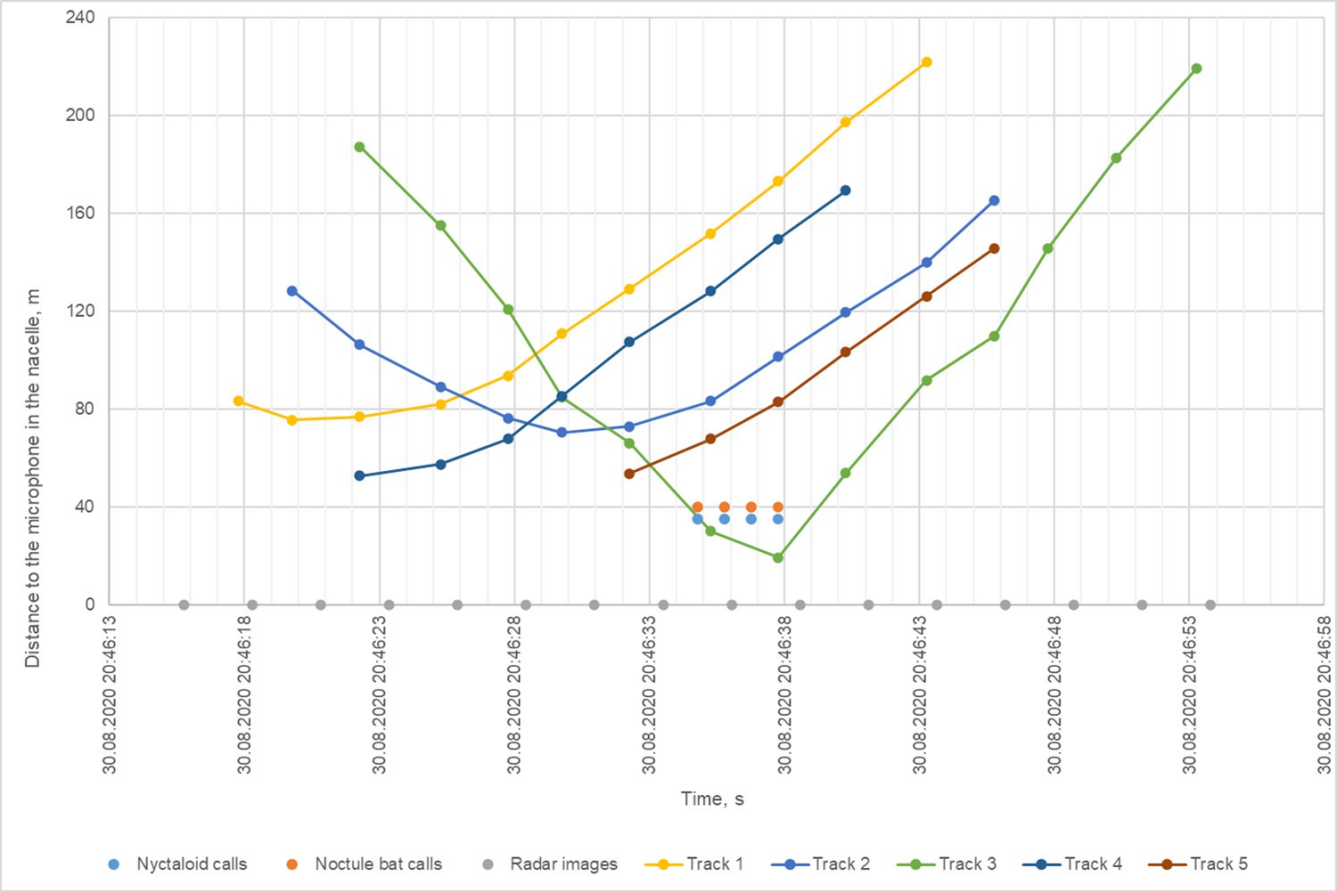
Distance over time diagram of the aligned typical bat event. Tracks are represented as a distance of a radar echo from the ABD at the timestamp of a corresponding radar image. Acoustic data is represented as points in time of the new aligned timestamp at the estimated detection distance of the bat detector for a corresponding frequency.

Following temporal alignment with the radar’s timeline, the timestamps of the recordings were shifted forward by 3 to 5 s, which was within the estimated worst-case of 16 s for the clock drift. For the previously mentioned typical bat event (Fig 9), the resulting aligned distance over time diagram for radar and acoustic data (see section "Selection and evaluation of data" for more details) is shown in Fig 10. In this bat event, only one track (track 3) has the potential to trigger the acoustic recordings as the timing of its approach and stay within the detection range of the ABD aligned with bat calls.

### Bat tracks

**Fig 11:**
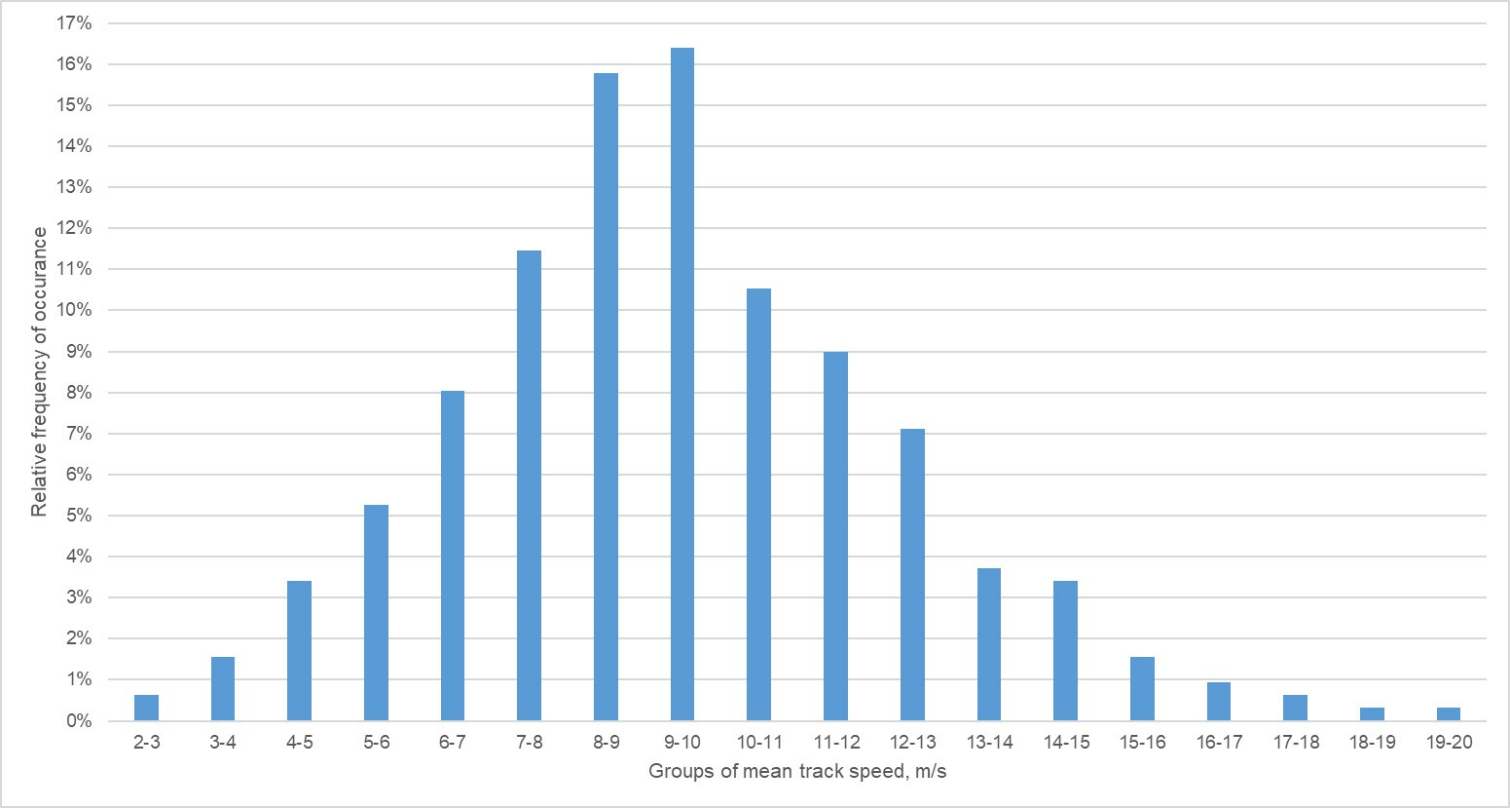
Relative frequency graph of the average track speed of the identified tracks (n = 323). Tracks with same boundary integer values were grouped together in one category.

The identified 323 tracks consisted of a mean of 5.9 radar echoes with a mean track length of 111.5 m (see S2 Table). The mean speed of the identified tracks is illustrated in Fig 11, where the most common mean track speed lies within a range of 9–10 m/s. It should be noted that any changes of the bats’ altitude were not available and therefore neglected in the calculation.

For comparison, Bruderer and Popa-Lisseanu recorded an average ground speed of 13.5 m/s and an average vertical speed of 1.9 m/s for a *N*. *noctula* bat [44]. However, this speed was measured by releasing a previously captured individual and is likely to be not fully representative for the free-flying bats of the same species. O’Mara et al. [61] recorded mean airspeeds between 7.2 – 15.9 m/s and mean ground speeds between 6.7 – 18.6 m/s for *N*. *noctula bat*, which corresponds to more than 80% of our data set.

Of the 323 tracks, 77 (about 24%) crossed the circumference around the microphone in the nacelle with r = 40 m. However, of these, only 29 tracks could be directly associated with an acoustic recording and the remaining 48 were not recorded by the ABD. This can be explained by considering how much bigger the radar detection volume is than the ABD volume (see Fig 6). A further 76 tracks (24%) had the potential to be associated with the acoustic recordings but did not cross the r = 40 m circumference.

Most of the identified tracks (95%) can be described as being straight or having slightly curved trajectories with an easily identifiable linear movement, corresponding to the general description of the common noctule bat’s flight style [62]. However, considering the refresh rate of the radar’s plan position indicator display of 2.5 s, it is virtually impossible to capture more detailed track trajectories, e.g., zigzag movement or other abrupt directional movement changes.

To avoid possible miscounts of diurnal birds’ tracks, this study was limited to only consider night time (see section "Data selection and evaluation"). However, we cannot completely exclude the possibility of some identified tracks belonging to diurnal birds. E.g., some bird tracks might be related to the roosting flights of swifts at night. According to the calculations based on the average body mass [25, 58], the estimated magnitude of a swift’s RCS is similar to that of noctule bats. Studies of the flight behavior of swifts [63–65] indicate that most of their activity is at altitudes above 1000 m, which is much higher than the altitude covered by our radar beam (see Fig 2). Therefore, roosting tracks of swifts should not be visible in our radar images. Another source of miscounted tracks is related to the nocturnal activity of some diurnal bird species during migration, and occasionally between migration periods. Several reports of nocturnal activity of songbirds can be found in the literature [32, 66–70]. Moreover, a number of songbird species was recorded in the proximity of the area of the studied wind park during the pre-construction species conservation assessment of the avifauna [71]. However, considering the bat-friendly weather conditions during the bat events, the mean speed of the identified tracks, and the acoustic evidence of bat presence during the track observations, we conclude that most of the identified tracks belong to bats. This is especially the case for the period from approximately mid-June until the end of July when most of the locally present bird species are not migrating and are not frequently active at nighttime.

### Comparison of acoustic and radar data

Acoustic and radar data for bat events were independently investigated for their similarity. Five correlations were tested:

- the correlation between the number of bat call recordings vs. the number of all tracks;
- the correlation between the duration of the recordings per bat event vs. the number of all tracks;
- the correlation between the number of bat call recordings vs. the number of tracks with the potential to be associated with bat calls;
- the correlation between the duration of the recordings per bat event vs. the number of tracks with the potential to be associated with bat calls;
- the correlation between the length of the acoustic sequences and the number of tracks with the potential to be aligned to these sequences.

All five correlations ranged from very weak to no association.

**Fig 12:**
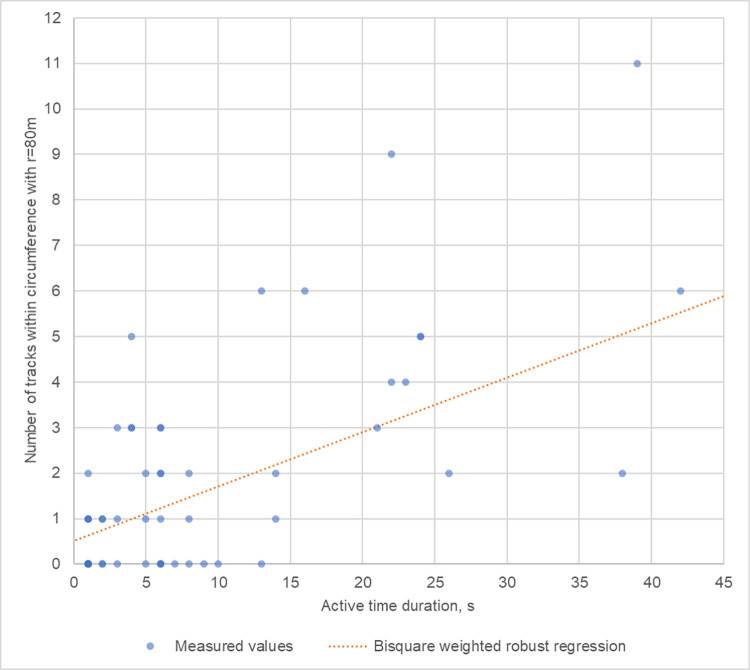
The number of tracks within outer circumference during active time of a bat event. Orange dashed line illustrates the robust regression line ***y* = 0.1169∙*x*+0.5146**.

Fig 12 depicts the Bisquare weighted robust regression (b = 0.1196, 95% CI: 0.0780, 0.1611; p = 0.000005) results of the duration of the active time in a bat event vs. the number of tracks found within r = 80 m during the active time. The longer the active time, the higher the likelihood of detecting more tracks near the WT’s nacelle. More data is needed to finetune this linear relationship, but it is worth noting that the regression line in Fig 12 predicts about 1 track within 80 m from the ABD within 5 s of active time. Incidentally, this ratio, 5 to 1, is also used by some researchers and consultants to calculate the bat activity metric "bat pass" using acoustics [19, 72, 73].

### Estimated RCS

**Fig 13:**
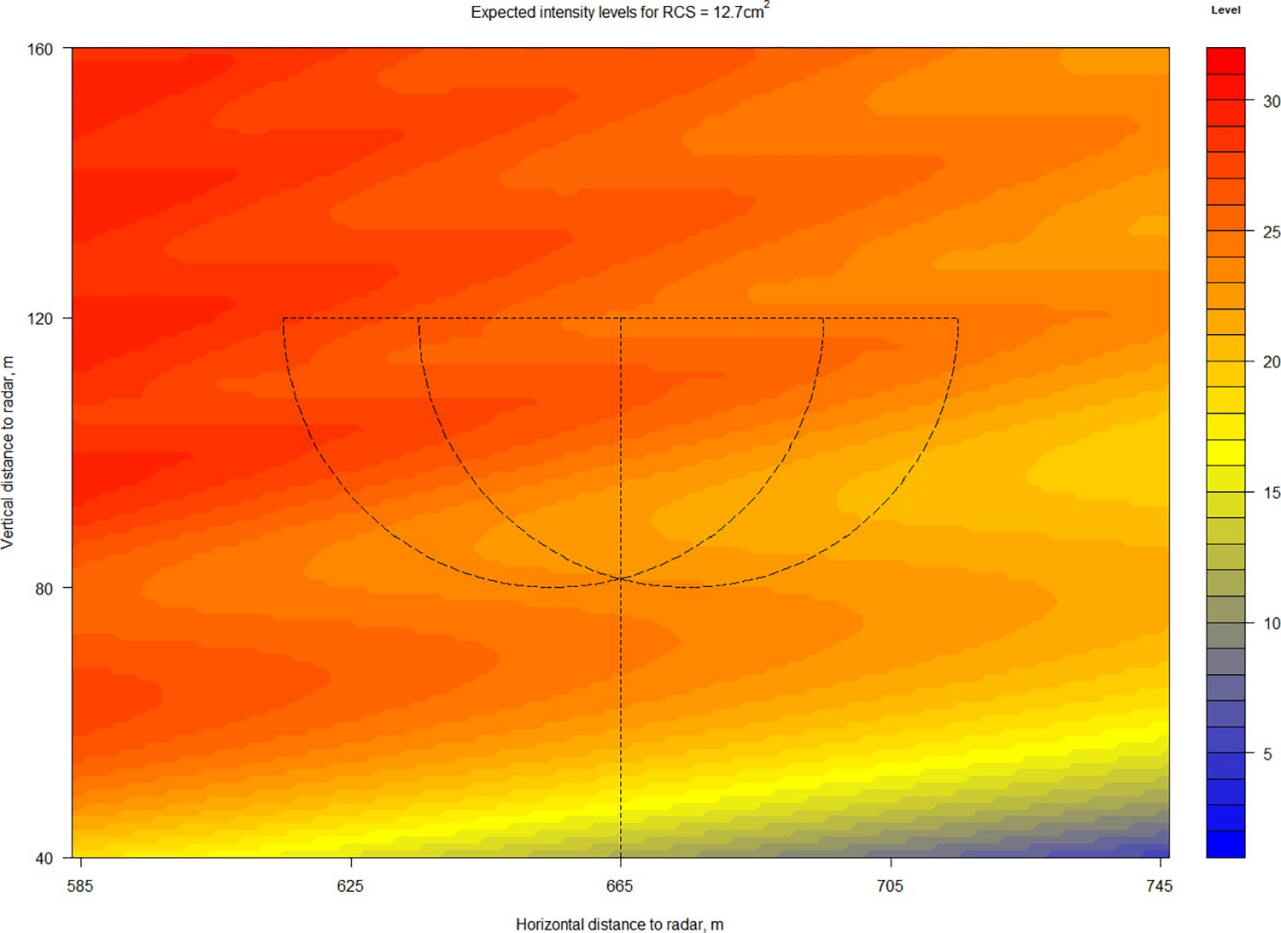
Expected intensity levels for an object with an RCS of 12.7cm^2^ near a wind turbine. Expected intensity levels calculated from simulated reflected power using a robust regression line. Two half-circles (r = 40m) represent the two extreme positions (the closest to and furthest from the radar) of the simplified detection volume of the ABD for bat calls with frequencies f ≤ 20 kHz. The vertical line gives reference to the tower position and height of the WT.

The study of radar echo intensities focused on the tracks with the potential to be associated with the acoustic recordings, specifically on the radar echoes within 40 m of the location of the ABD. Fig 13 depicts the predicted intensity levels of our analytical simulation model for an object with an RCS of 12.7 cm^2^, which corresponds to a fully exposed noctule bat [49] near the WT’s nacelle. The half circles in Fig 13 represent the closest and the furthest possible positions of the detection volume of the ABD. The ABD microphone was installed facing downwards, towards the back of the nacelle, and the nacelle position was assumed to be aligned with the direction of the wind. The wavy pattern of the level distribution originates from the shape of the radar detection volume (see Fig 2). Here, the intensity levels are more spread along the main beam direction than at the lower detection edge of the simulated detection volume due to the influence of the clutter shielding fence.

**Fig 14:**
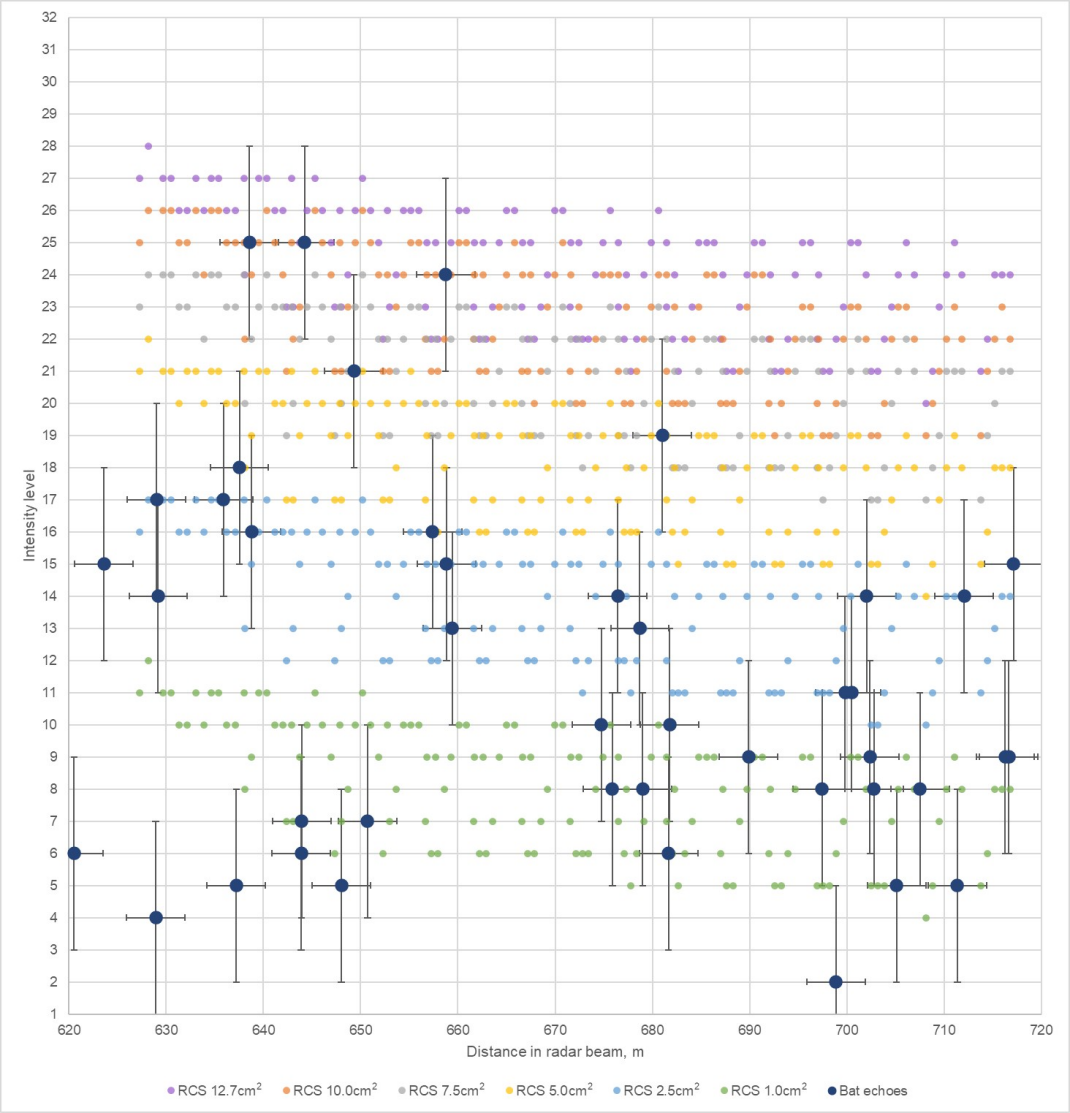
Expected and measured intensity levels within the detection volume of the bat detector. Simulated values sampled in 5 m intervals for vertical and horizontal distances. Horizontal whiskers indicate the inaccuracy of the measurement position. Vertical whiskers consider the measurement uncertainty of ± 3 intensity levels (± 2 dBm).

To keep the results of measurements and simulations comparable (in Fig 14), we remapped the vertical and horizontal coordinates (X,Y) of the simulated values found in the acoustic detection volumes (r = 40 m) to the distance of the radar beam. The result of this mapping for objects with an RCS of 10.0, 7.5, 5.0, 2.5 and 1.0 cm^2^ are depicted in Fig 14. For comparison, the measured intensity levels of radar echoes from the tracks with the potential to be associated with acoustic recordings were also plotted in Fig 14 over their corresponding distance of the radar beam.

An overall decreasing trend for expected intensity levels over the increasing distance in the radar beam can be observed in Fig 14 for each of the simulated RCS values, while no such trend can be seen for the measured radar echo intensity levels. Considering the measurement uncertainty of ± 3 intensity levels (± 2 dBm), several high intensity radar echoes can be aligned with RCSs between 5.0–12.7 cm^2^. Most radar echoes align with RCSs between 1.0–5.0 cm^2^. The RCS values of *N*. *noctula* recorded during the study with our radar setup are lower than anticipated. However, compared to the bat model’s RCS of 12.7cm^2^ from [49], which was calculated in stationary conditions being fully exposed to the antenna beam, our measured RCSs were calculated in-flight. In addition, it is likely that the bat model presented in [49] is of an individuum with a body mass at the higher end of the typical body mass of the *N*. *noctula* and that the *N*. *noctula* bats we recorded were under-exposed and generally of a more diverse mass. For comparison, the RCS of *T*. *brasiliensis* (with a typical body mass between 7 and 12 g; RCS between 1.49 – 2.78 cm^2^ [48]) as well as the values of RCSs for other stationary recorded wildlife [26, 74–78] in the X-band showed great variation depending on the angle of incidence to the antenna beam, which further supports our results.

### Implications of this study

This study is an important step towards the acceptance and adoption of X-band radars in horizontal scanning configurations for large scale bat monitoring applications. The detection volume is considerably increased compared with an ABD, covering the full rotor-swept area of several modern WTs. Detection in vertical space over large distances, even without the information on target height, can open up new opportunities, e.g., for validating currently used curtailment algorithms for wind turbines or to get deeper insights into bats’ flight patterns within wind parks. Whilst we focus our validation for this study on approximately 665 m, it is clear, that for smaller distances the resolution would increase and even smaller objects could be reliably detected. Increasing the antenna rotation rate results in a higher number of images, but would allow for more precise bat tracking. An X-band radar is also a relatively inexpensive monitoring solution compared to specialized bird detection radars, making it more accessible for researchers.

## Conclusions

In conclusion, a portable pulse X-band radar is a suitable tool to detect and track individual bats, particularly the common noctule bats (*Nyctalus noctula*). In this study, we investigated systematically and validated the detection of *N*. *noctula* bats in the proximity of an onshore wind turbine at a distance of 665 m from the radar. Applying a structured methodology for the data selection process and the manual tracking, we found bat tracks for 96% of our bat events. For 83% of the bat events, data association between bat call sequences and bat tracks were possible. Although we could not completely rule out the presence of birds in our dataset, most tracks must belong to bats since the extracted values of parameters from the tracks were consistent with the values found in the literature.

## Supporting information

S1 Table Results of preliminary study of echo intensity. (XLSX)

S2 Table (XLSX)

S1 File Figures of bat events. (ZIP)
