# Supplementary figures and images for: Detection and validation of common noctule bats (Nyctalus noctula) with a pulse radar and acoustic monitoring in the proximity of an onshore wind turbine
Source: PLoS One. 2024 Jun 12;19(6):e0299153. doi: 10.1371/journal.pone.0299153 (PMC11168679; doi:10.1371/journal.pone.0299153)

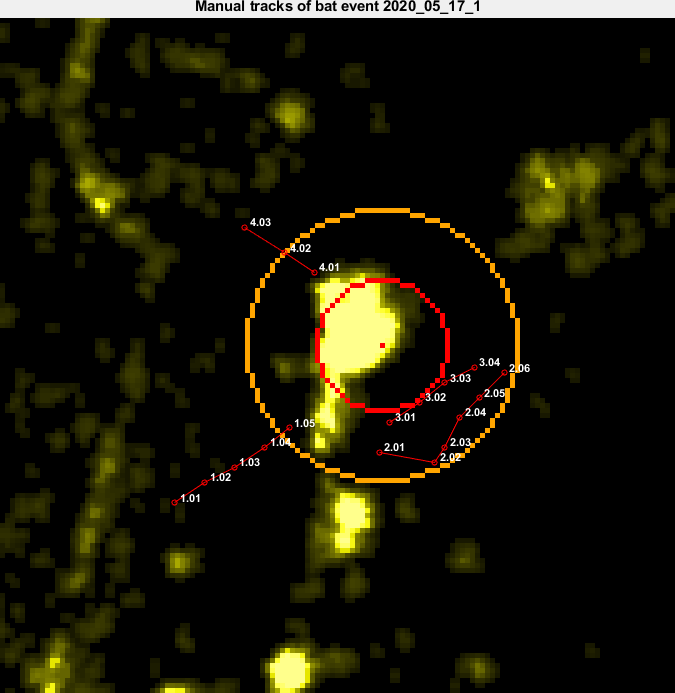

Supplement: S1 File — (ZIP) [file pone.0299153.s003.zip › Manual_tracks_2020_05_17_1.png]

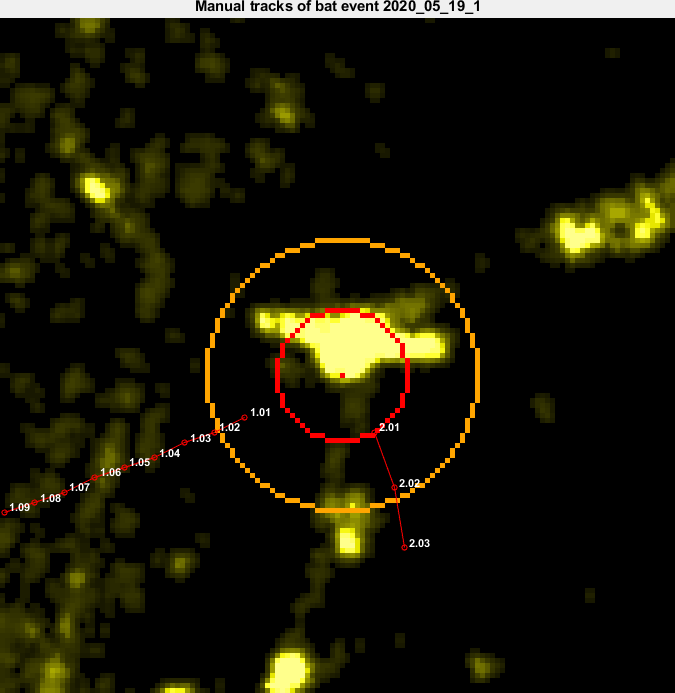

Supplement: S1 File — (ZIP) [file pone.0299153.s003.zip › Manual_tracks_2020_05_19_1.png]

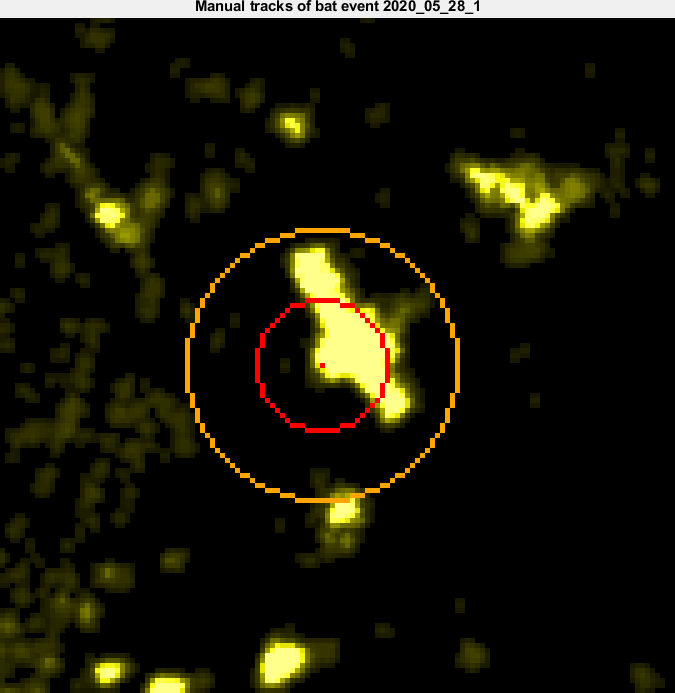

Supplement: S1 File — (ZIP) [file pone.0299153.s003.zip › Manual_tracks_2020_05_28_1.png]

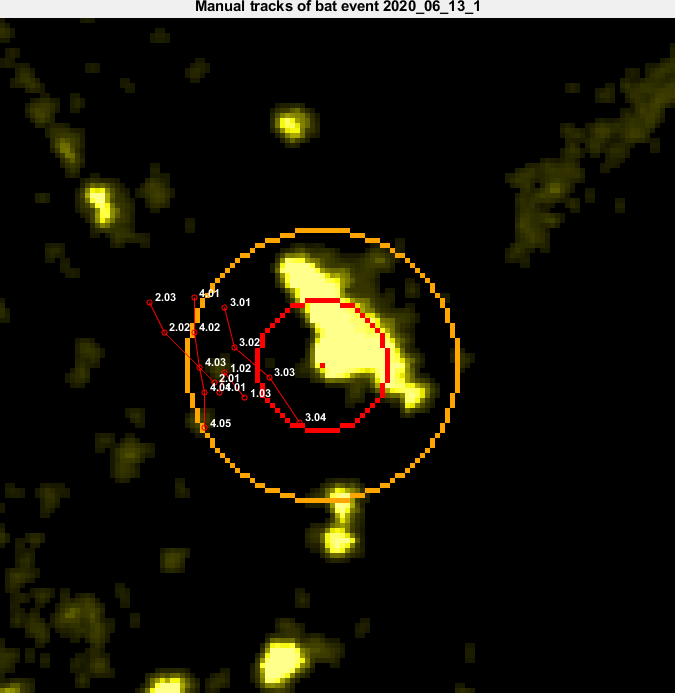

Supplement: S1 File — (ZIP) [file pone.0299153.s003.zip › Manual_tracks_2020_06_13_1.png]

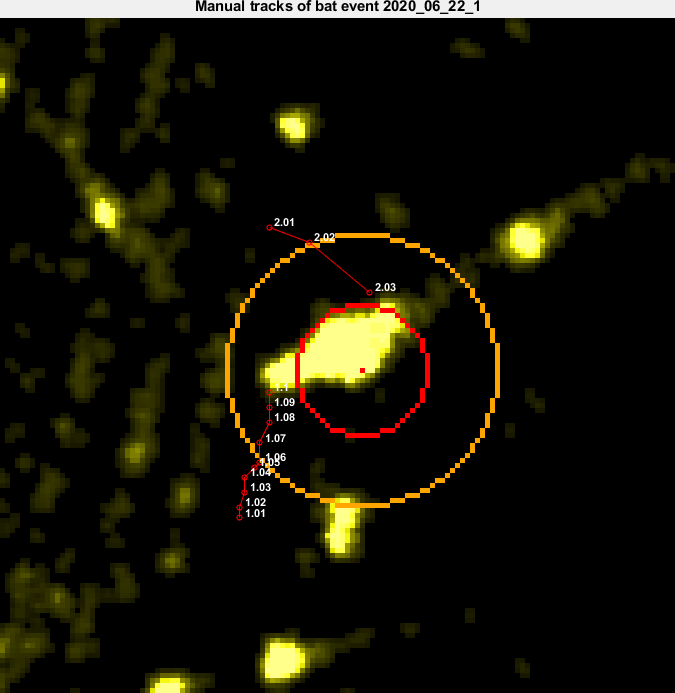

Supplement: S1 File — (ZIP) [file pone.0299153.s003.zip › Manual_tracks_2020_06_22_1.png]

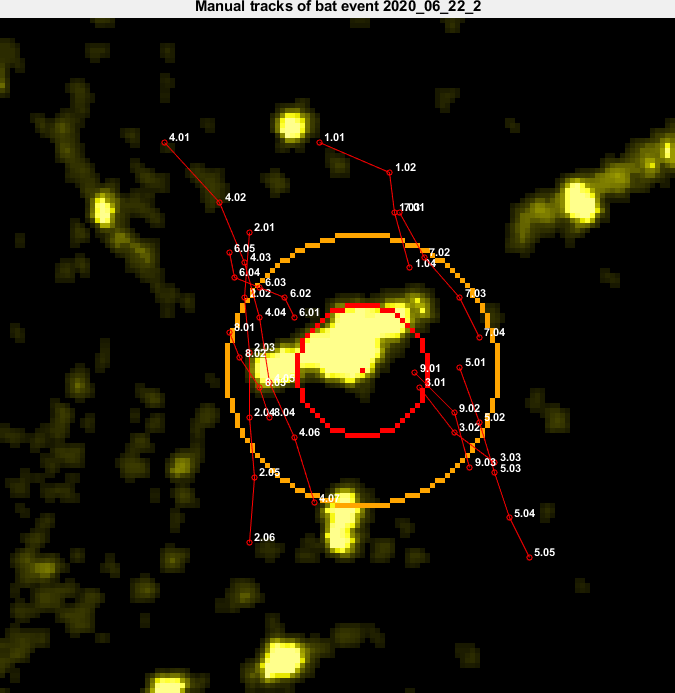

Supplement: S1 File — (ZIP) [file pone.0299153.s003.zip › Manual_tracks_2020_06_22_2.png]

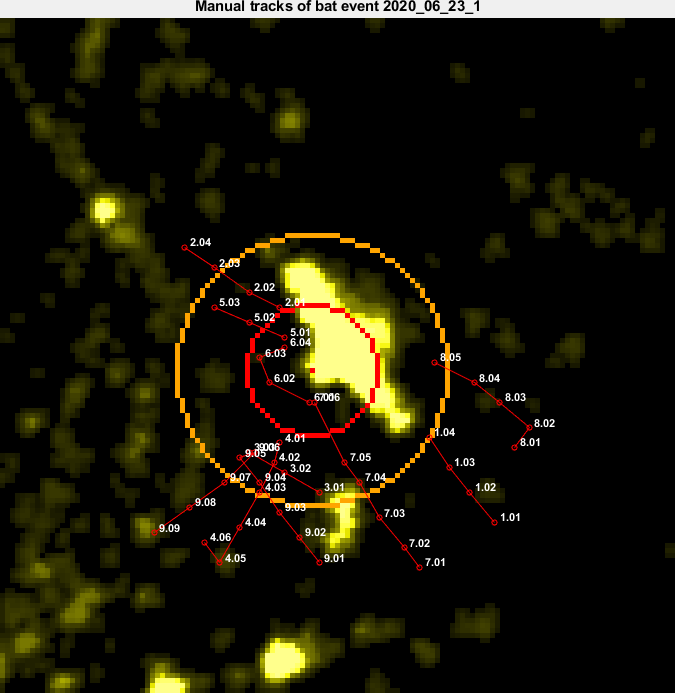

Supplement: S1 File — (ZIP) [file pone.0299153.s003.zip › Manual_tracks_2020_06_23_1.png]

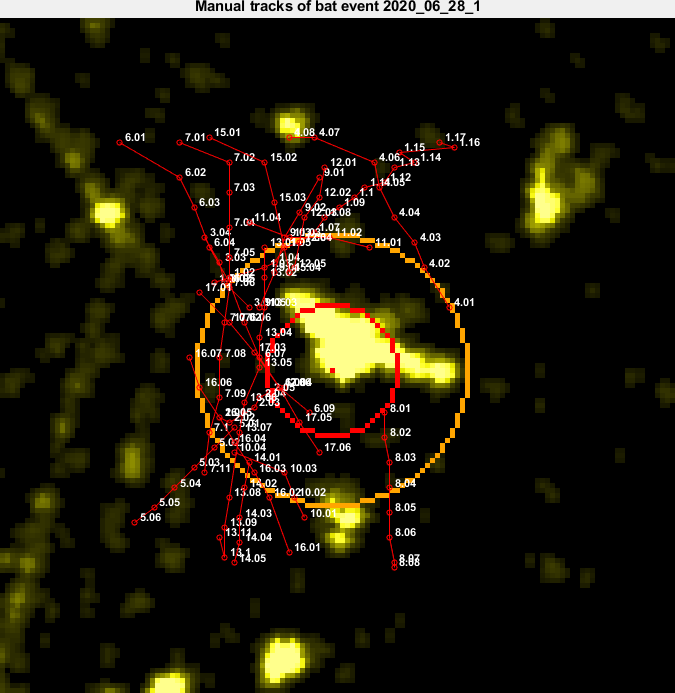

Supplement: S1 File — (ZIP) [file pone.0299153.s003.zip › Manual_tracks_2020_06_28_1.png]

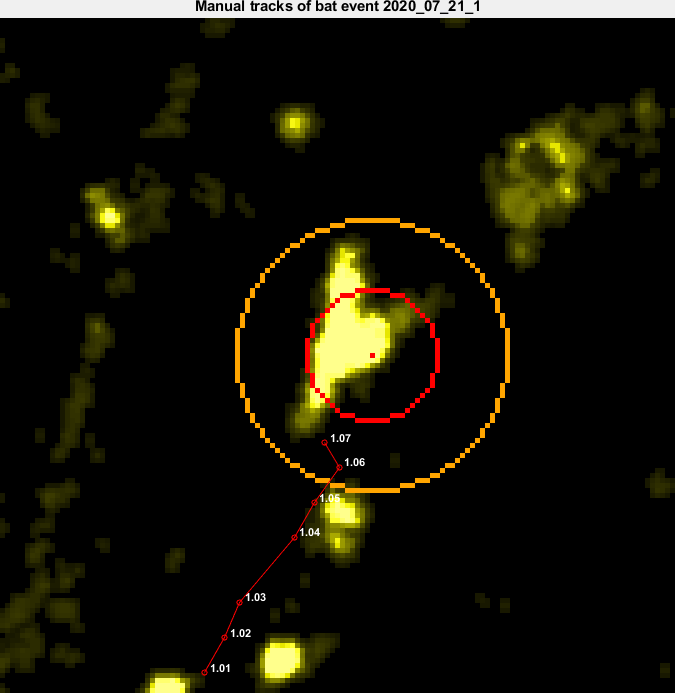

Supplement: S1 File — (ZIP) [file pone.0299153.s003.zip › Manual_tracks_2020_07_21_1.png]

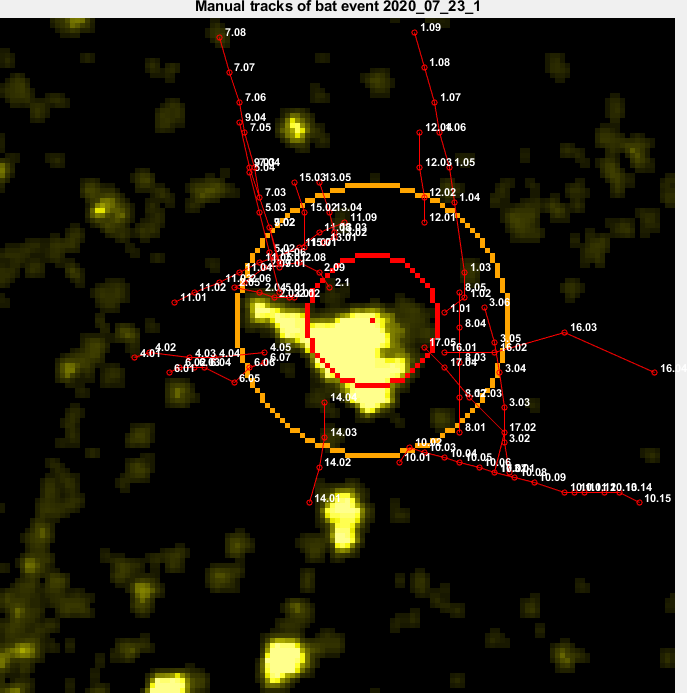

Supplement: S1 File — (ZIP) [file pone.0299153.s003.zip › Manual_tracks_2020_07_23_1.png]

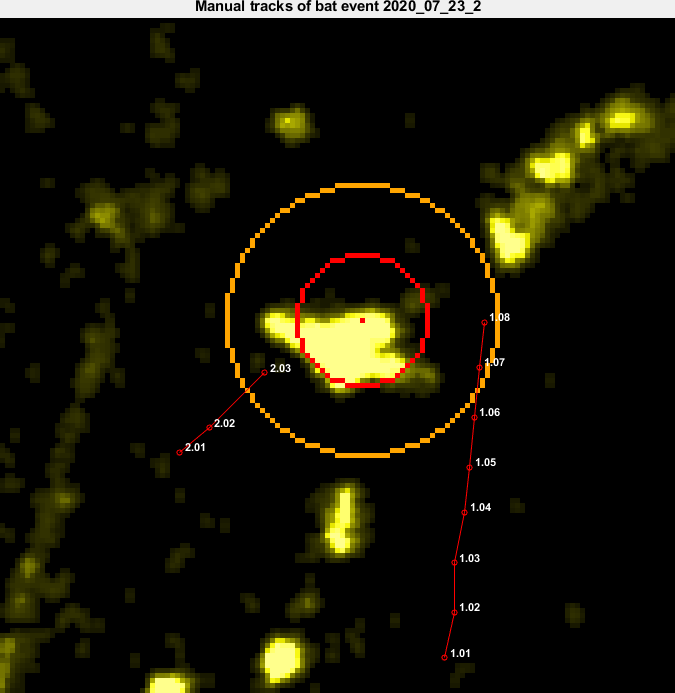

Supplement: S1 File — (ZIP) [file pone.0299153.s003.zip › Manual_tracks_2020_07_23_2.png]

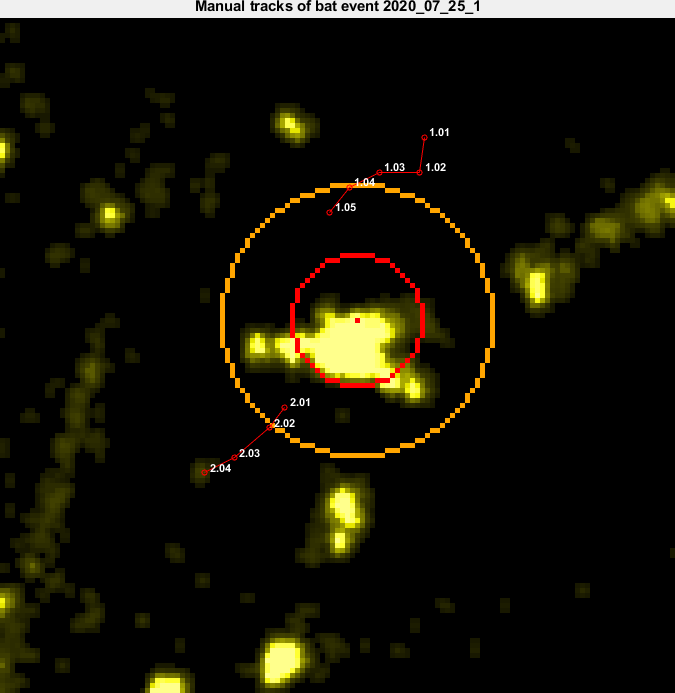

Supplement: S1 File — (ZIP) [file pone.0299153.s003.zip › Manual_tracks_2020_07_25_1.png]

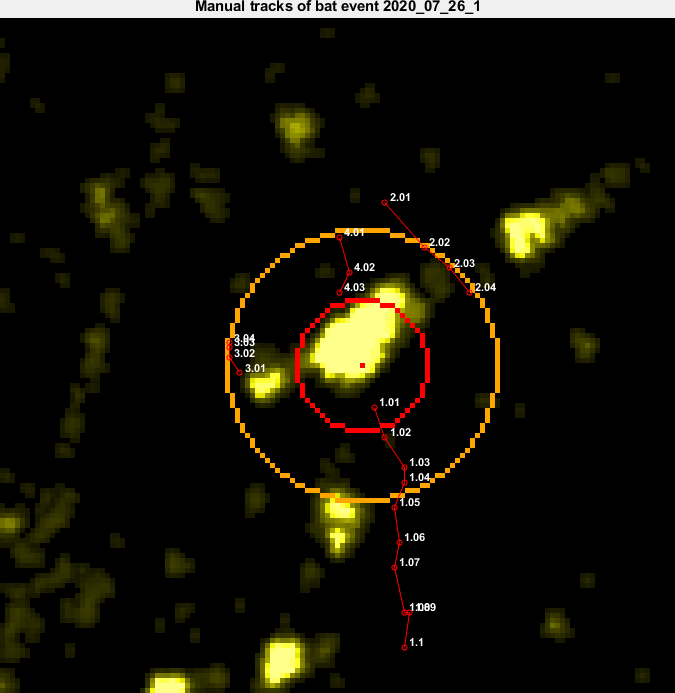

Supplement: S1 File — (ZIP) [file pone.0299153.s003.zip › Manual_tracks_2020_07_26_1.png]

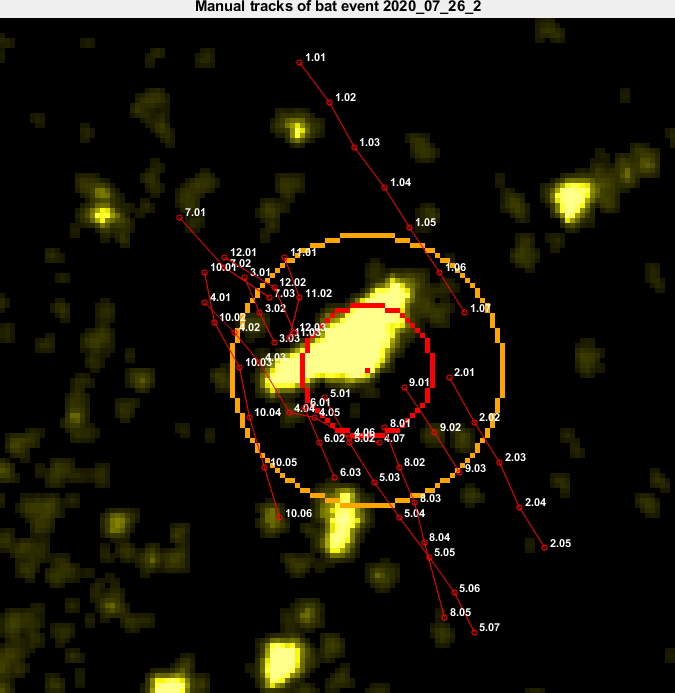

Supplement: S1 File — (ZIP) [file pone.0299153.s003.zip › Manual_tracks_2020_07_26_2.png]

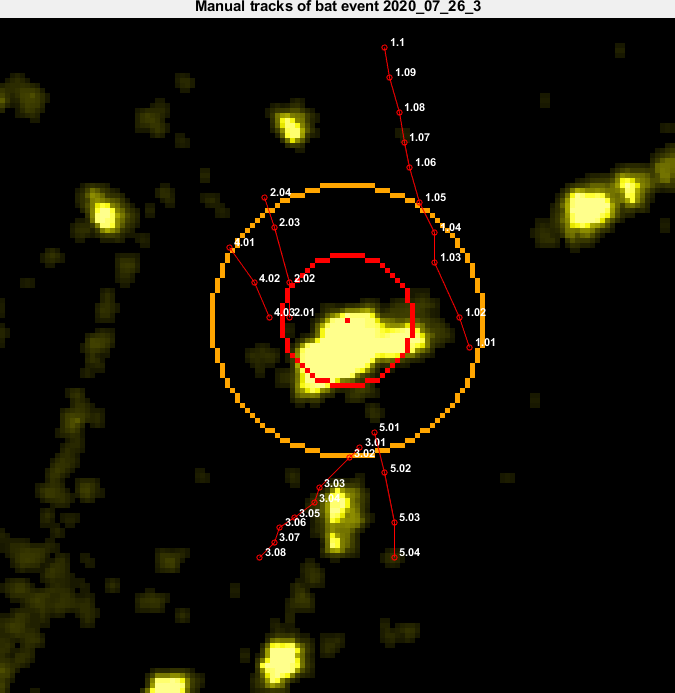

Supplement: S1 File — (ZIP) [file pone.0299153.s003.zip › Manual_tracks_2020_07_26_3.png]

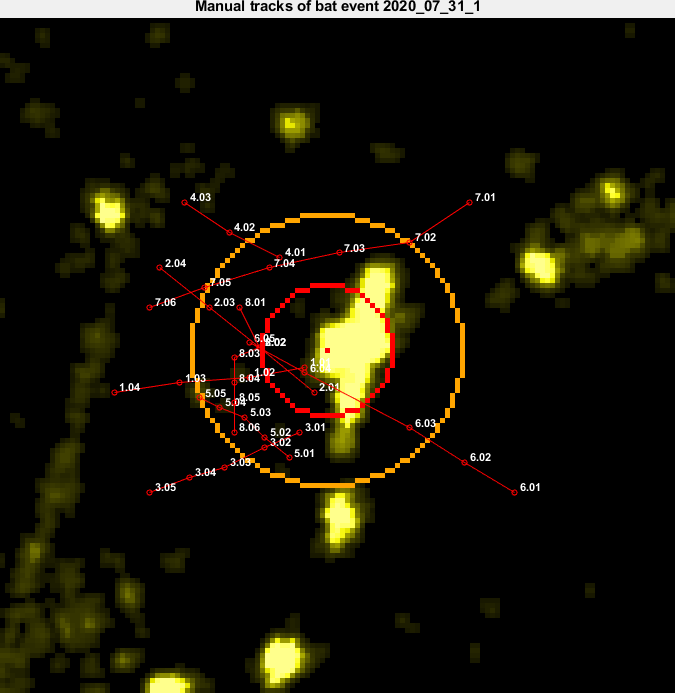

Supplement: S1 File — (ZIP) [file pone.0299153.s003.zip › Manual_tracks_2020_07_31_1.png]

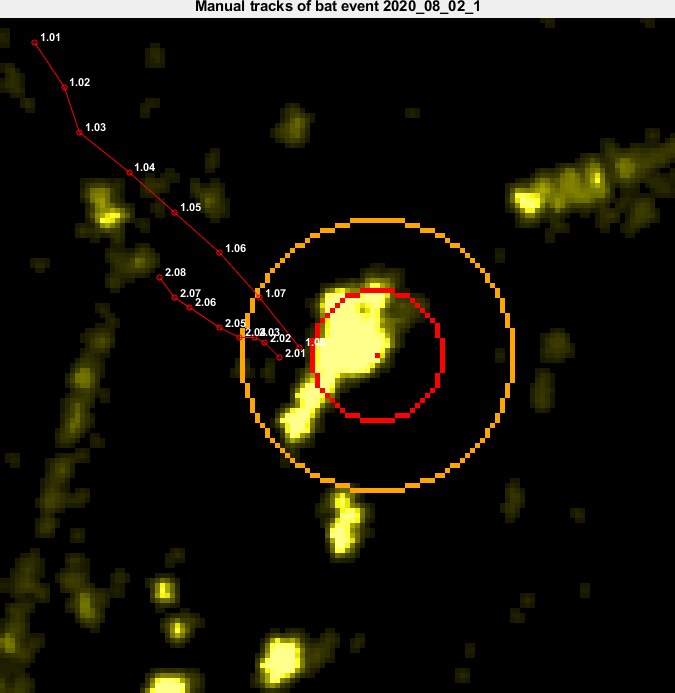

Supplement: S1 File — (ZIP) [file pone.0299153.s003.zip › Manual_tracks_2020_08_02_1.png]

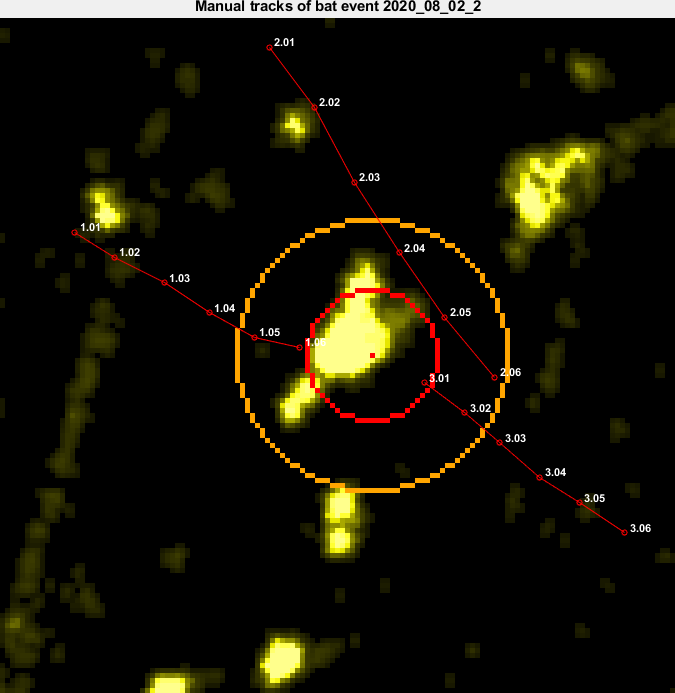

Supplement: S1 File — (ZIP) [file pone.0299153.s003.zip › Manual_tracks_2020_08_02_2.png]

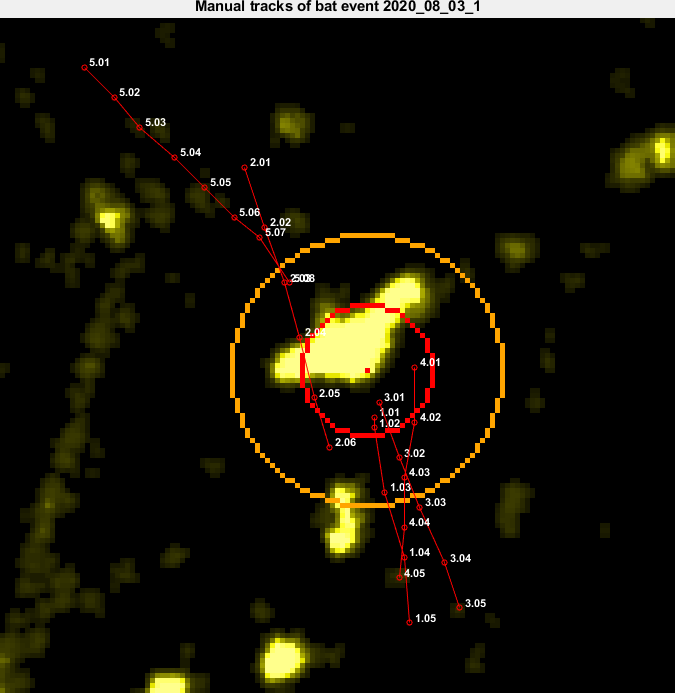

Supplement: S1 File — (ZIP) [file pone.0299153.s003.zip › Manual_tracks_2020_08_03_1.png]

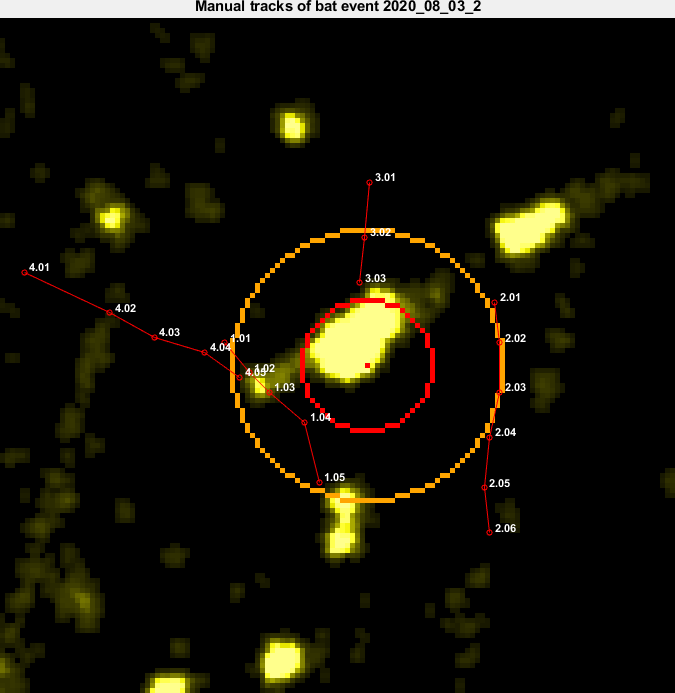

Supplement: S1 File — (ZIP) [file pone.0299153.s003.zip › Manual_tracks_2020_08_03_2.png]

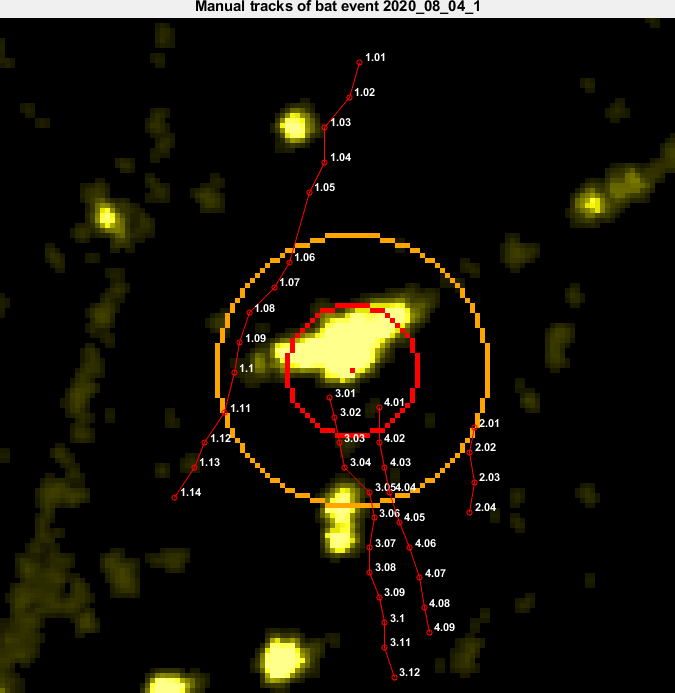

Supplement: S1 File — (ZIP) [file pone.0299153.s003.zip › Manual_tracks_2020_08_04_1.png]

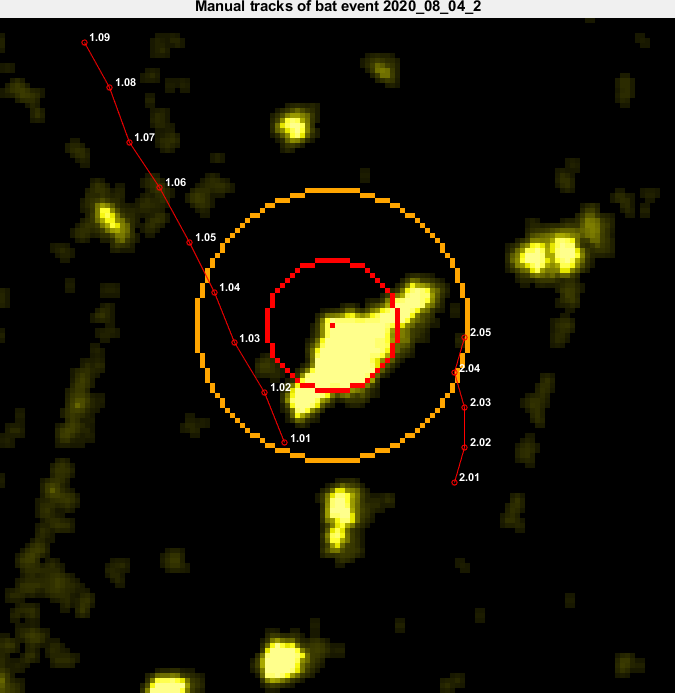

Supplement: S1 File — (ZIP) [file pone.0299153.s003.zip › Manual_tracks_2020_08_04_2.png]

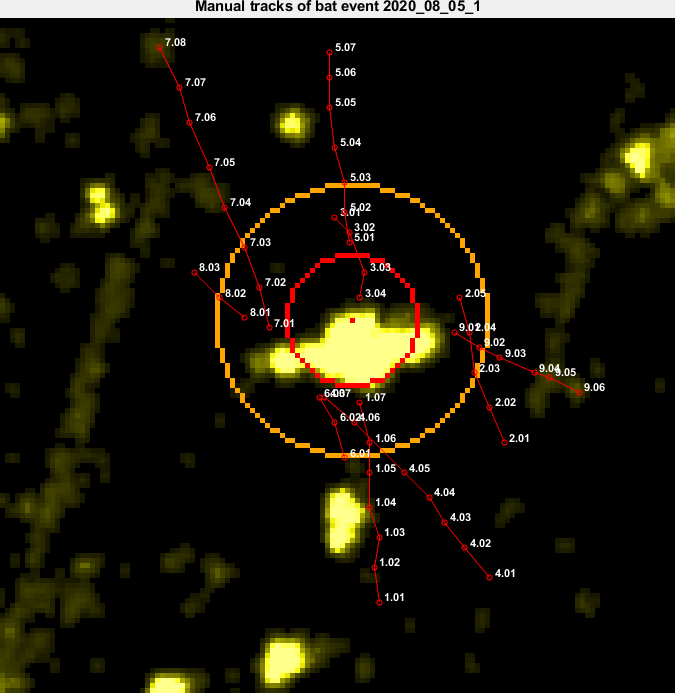

Supplement: S1 File — (ZIP) [file pone.0299153.s003.zip › Manual_tracks_2020_08_05_1.png]

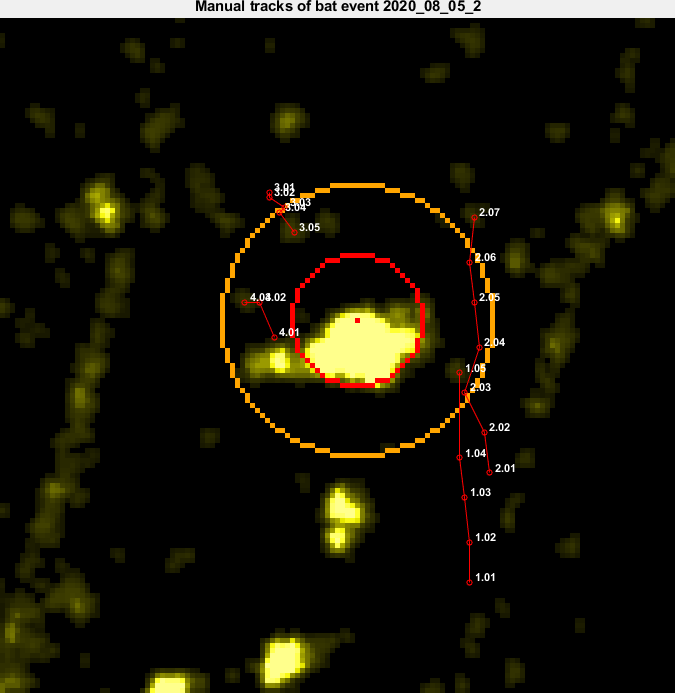

Supplement: S1 File — (ZIP) [file pone.0299153.s003.zip › Manual_tracks_2020_08_05_2.png]

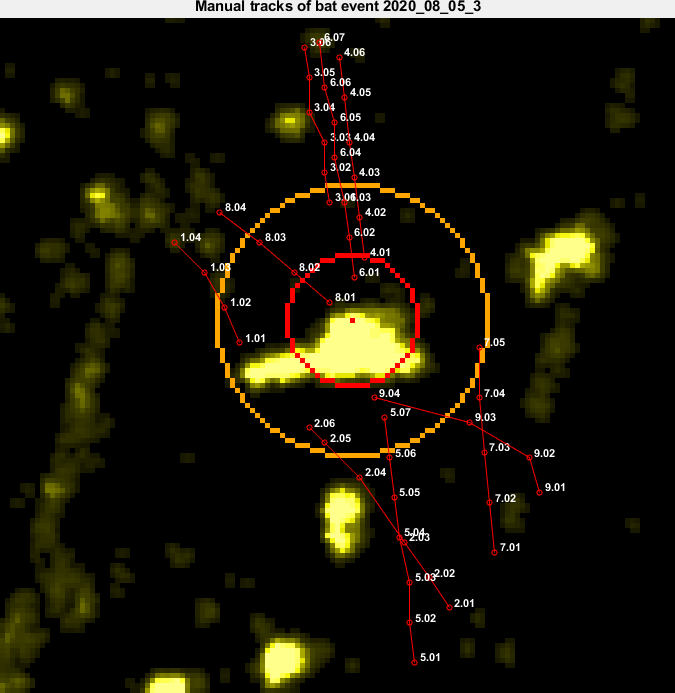

Supplement: S1 File — (ZIP) [file pone.0299153.s003.zip › Manual_tracks_2020_08_05_3.png]

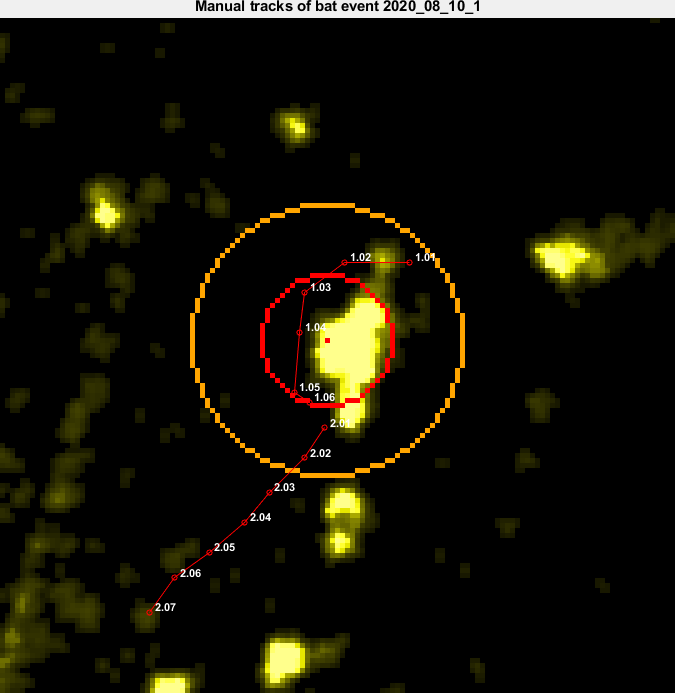

Supplement: S1 File — (ZIP) [file pone.0299153.s003.zip › Manual_tracks_2020_08_10_1.png]

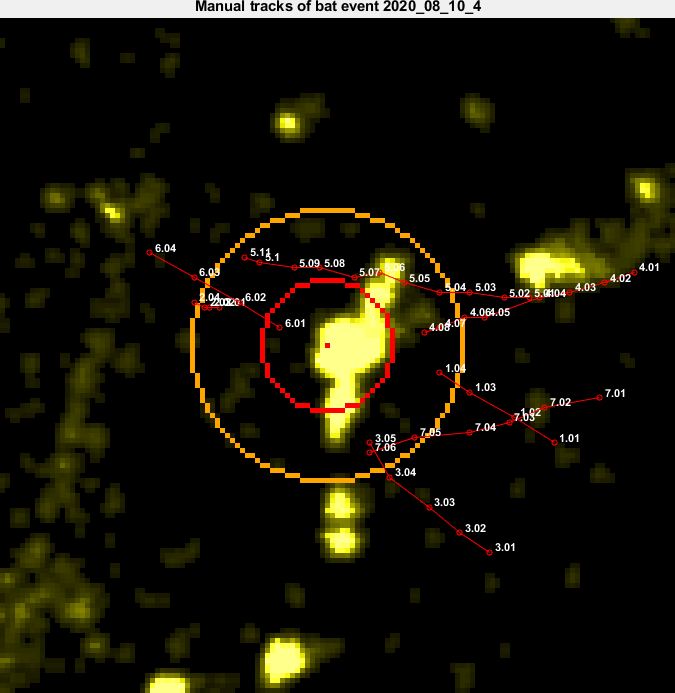

Supplement: S1 File — (ZIP) [file pone.0299153.s003.zip › Manual_tracks_2020_08_10_4.png]

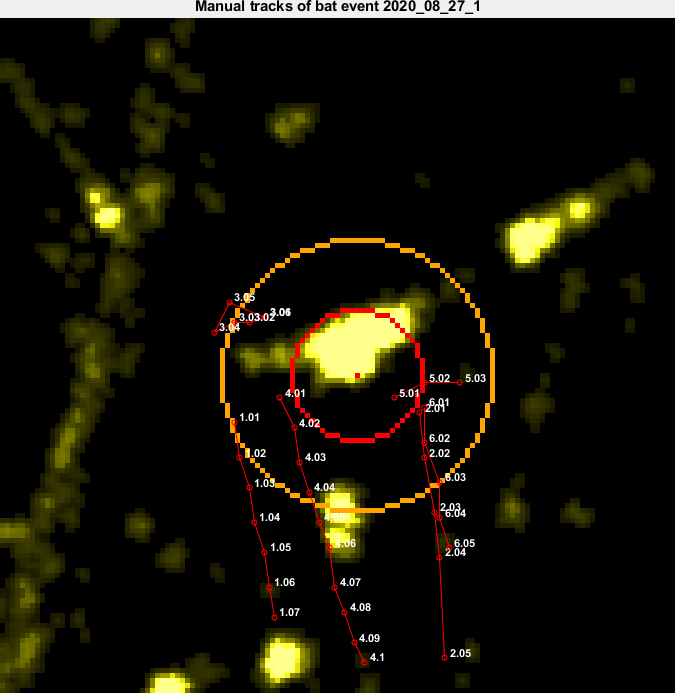

Supplement: S1 File — (ZIP) [file pone.0299153.s003.zip › Manual_tracks_2020_08_27_1.png]

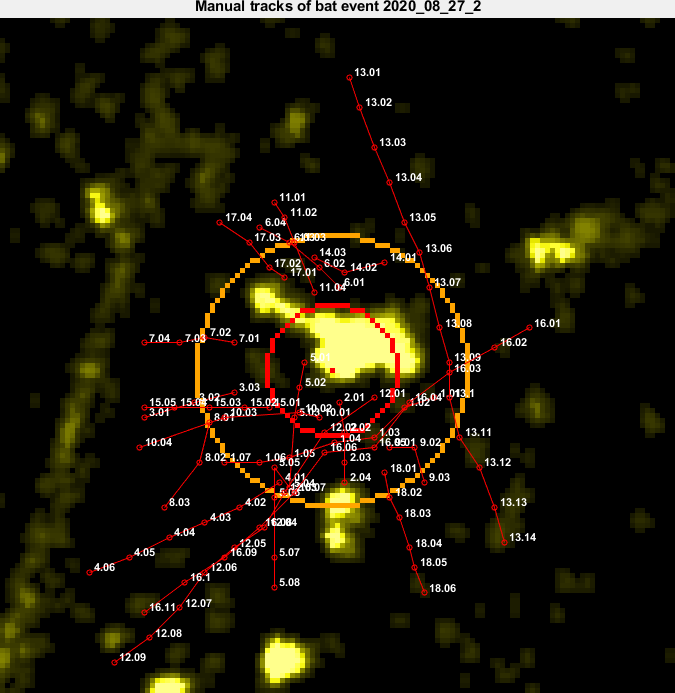

Supplement: S1 File — (ZIP) [file pone.0299153.s003.zip › Manual_tracks_2020_08_27_2.png]

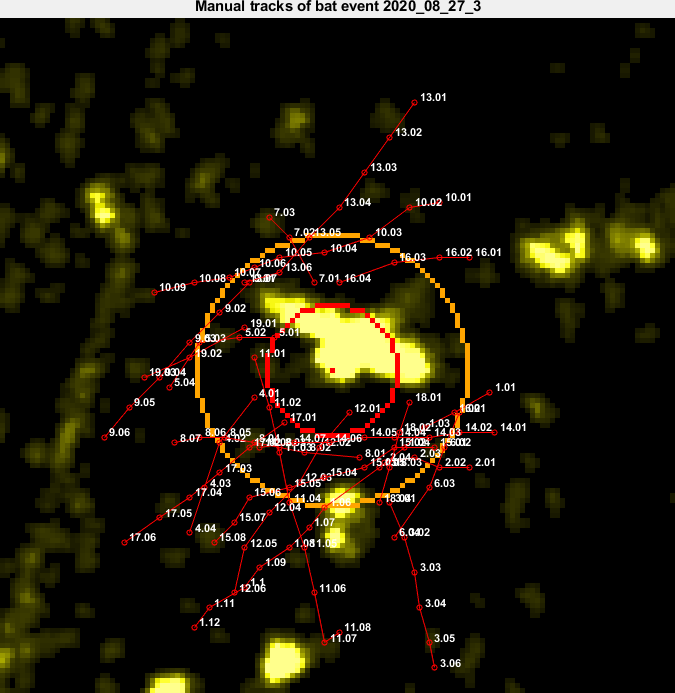

Supplement: S1 File — (ZIP) [file pone.0299153.s003.zip › Manual_tracks_2020_08_27_3.png]

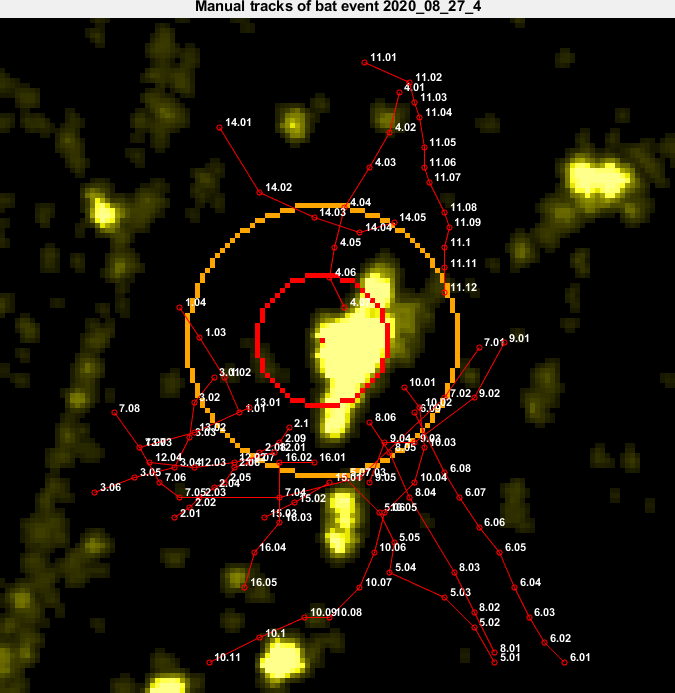

Supplement: S1 File — (ZIP) [file pone.0299153.s003.zip › Manual_tracks_2020_08_27_4.png]

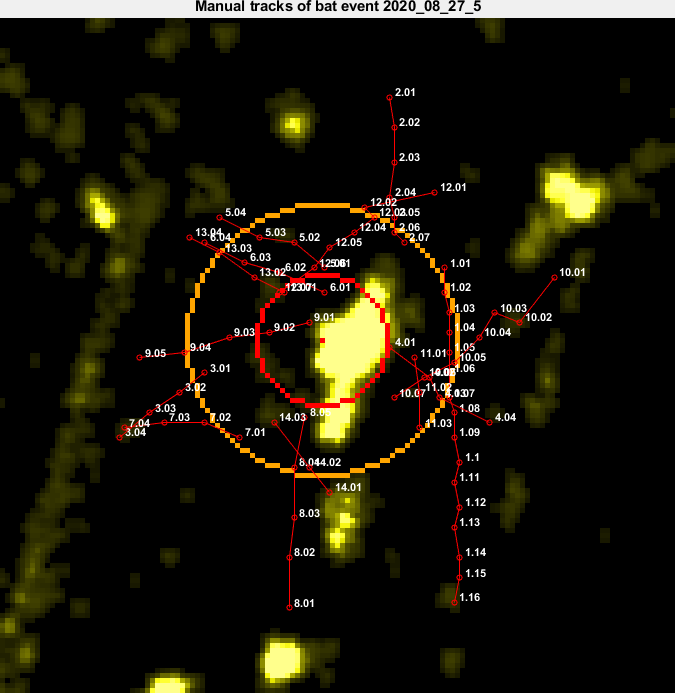

Supplement: S1 File — (ZIP) [file pone.0299153.s003.zip › Manual_tracks_2020_08_27_5.png]

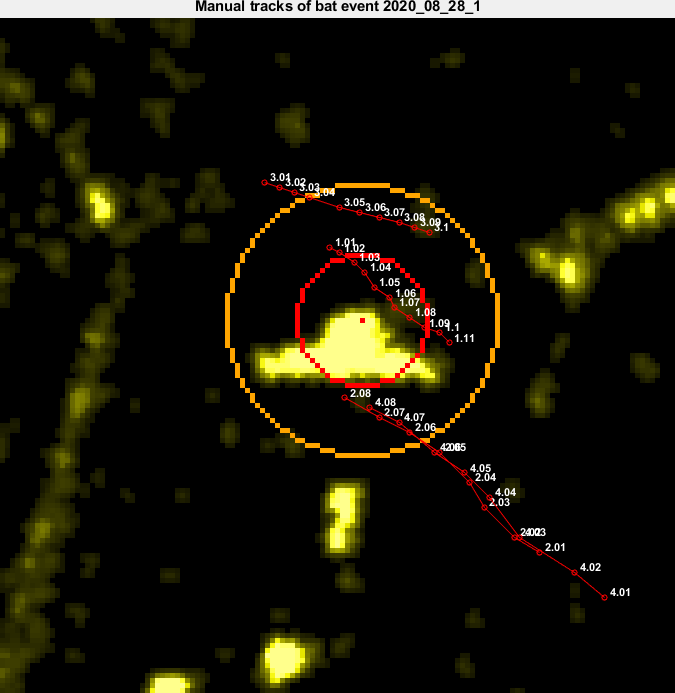

Supplement: S1 File — (ZIP) [file pone.0299153.s003.zip › Manual_tracks_2020_08_28_1.png]

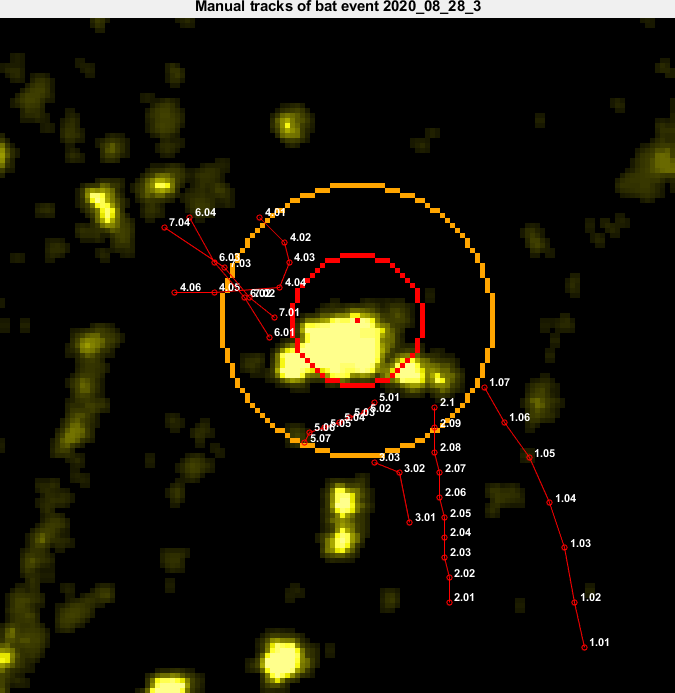

Supplement: S1 File — (ZIP) [file pone.0299153.s003.zip › Manual_tracks_2020_08_28_3.png]

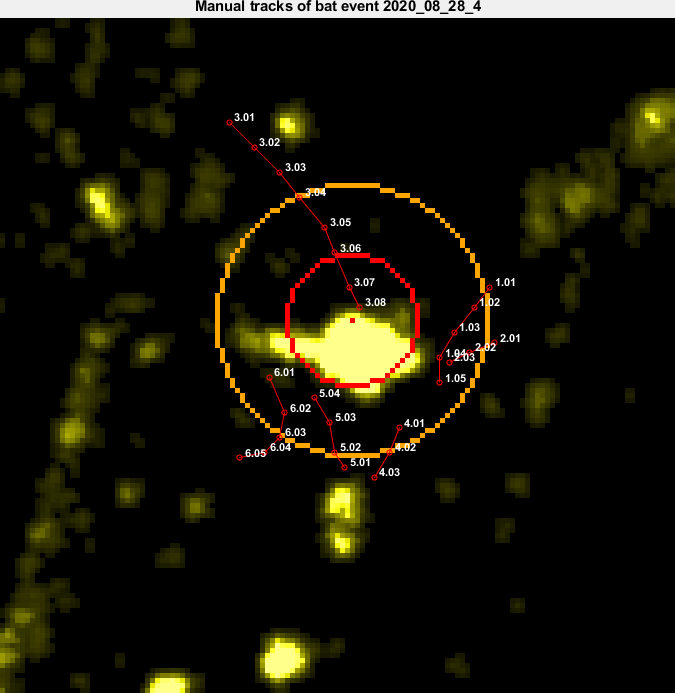

Supplement: S1 File — (ZIP) [file pone.0299153.s003.zip › Manual_tracks_2020_08_28_4.png]

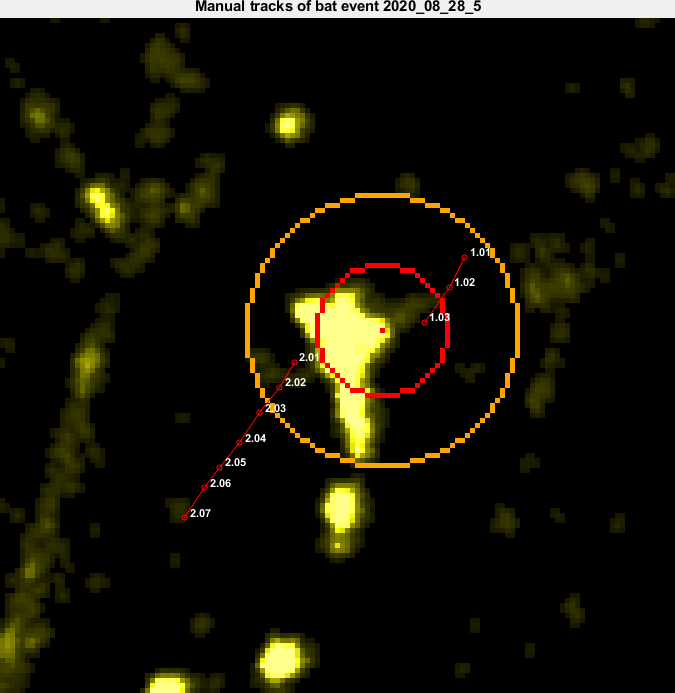

Supplement: S1 File — (ZIP) [file pone.0299153.s003.zip › Manual_tracks_2020_08_28_5.png]

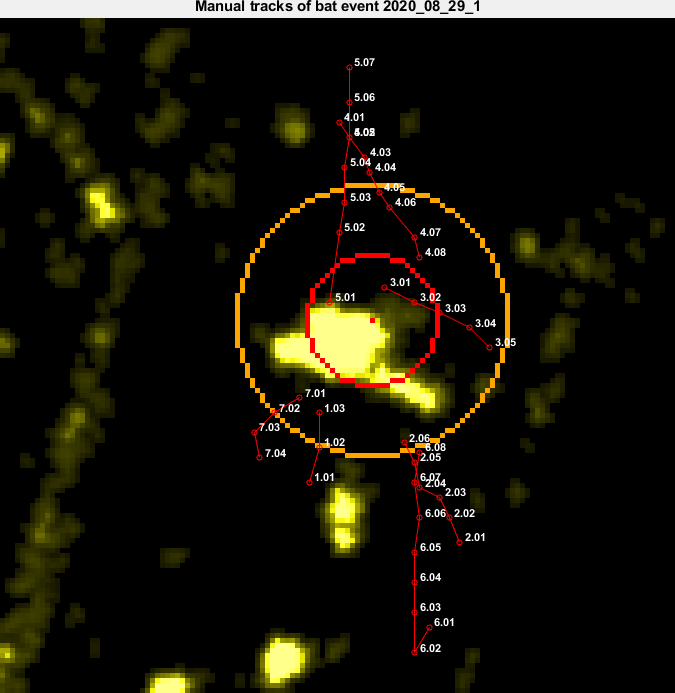

Supplement: S1 File — (ZIP) [file pone.0299153.s003.zip › Manual_tracks_2020_08_29_1.png]

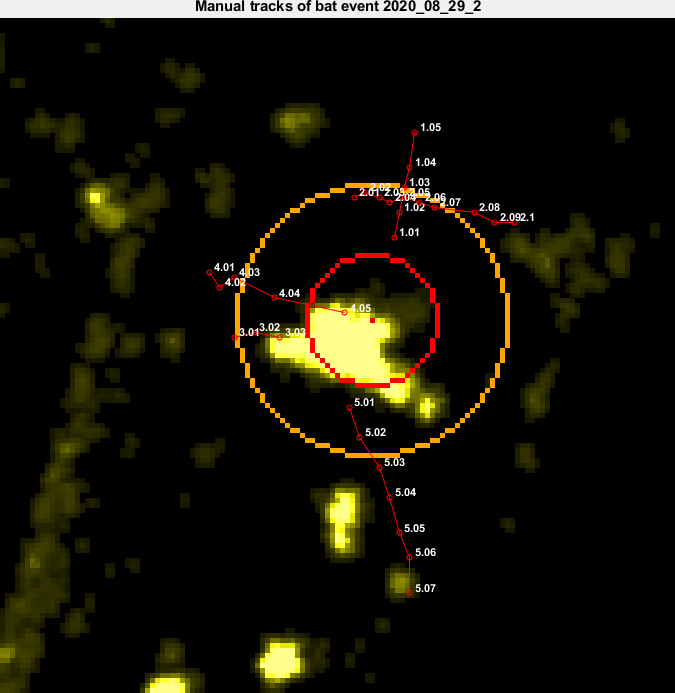

Supplement: S1 File — (ZIP) [file pone.0299153.s003.zip › Manual_tracks_2020_08_29_2.png]

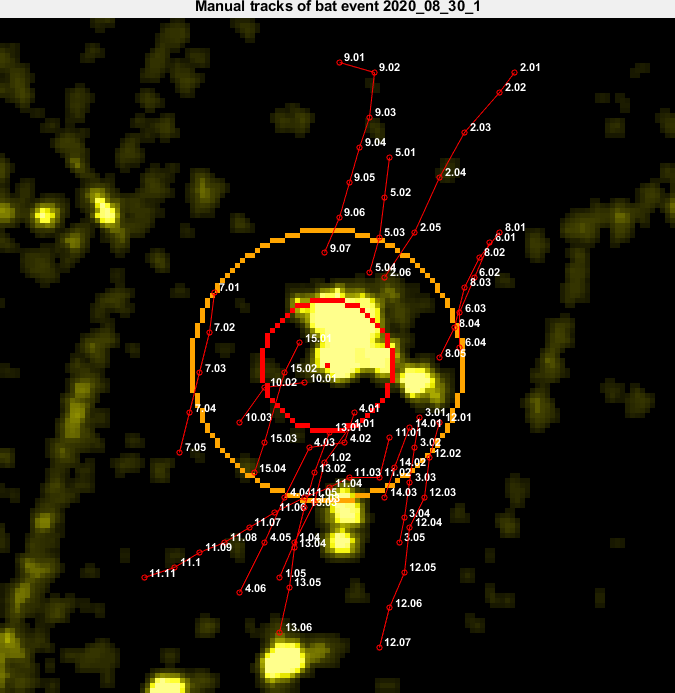

Supplement: S1 File — (ZIP) [file pone.0299153.s003.zip › Manual_tracks_2020_08_30_1.png]

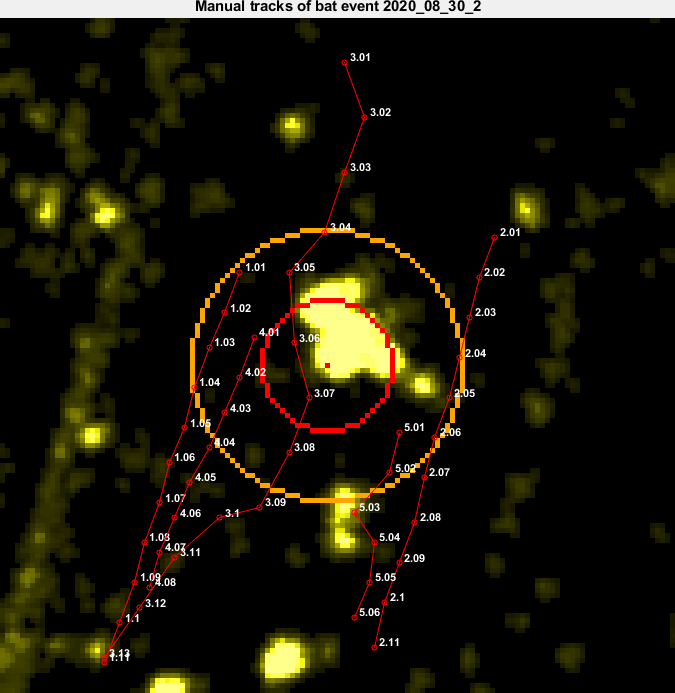

Supplement: S1 File — (ZIP) [file pone.0299153.s003.zip › Manual_tracks_2020_08_30_2.png]

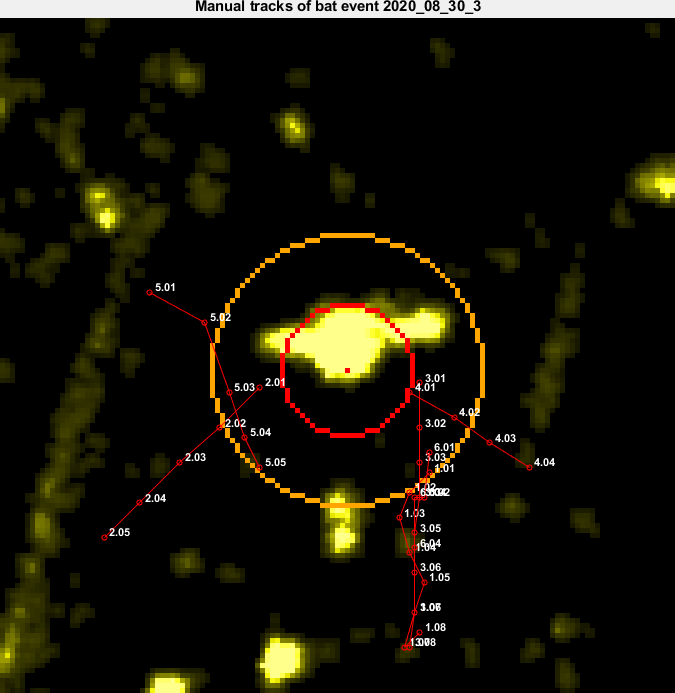

Supplement: S1 File — (ZIP) [file pone.0299153.s003.zip › Manual_tracks_2020_08_30_3.png]

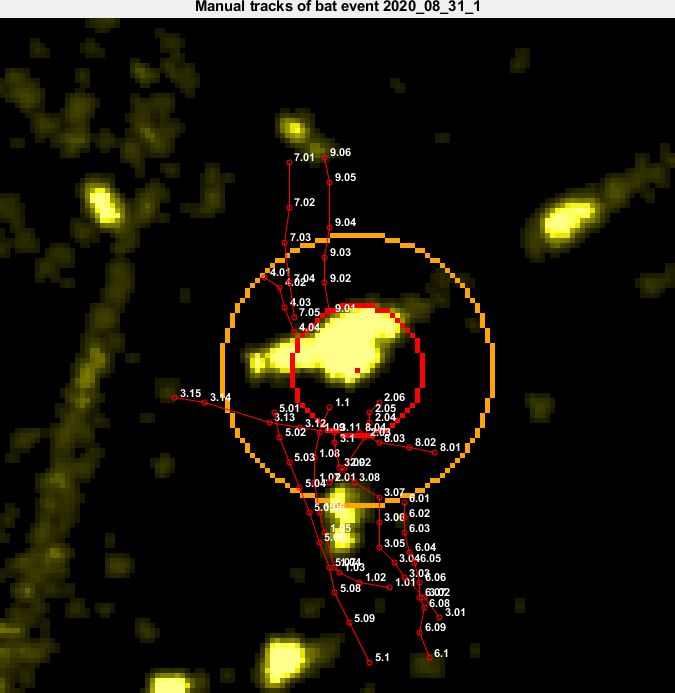

Supplement: S1 File — (ZIP) [file pone.0299153.s003.zip › Manual_tracks_2020_08_31_1.png]

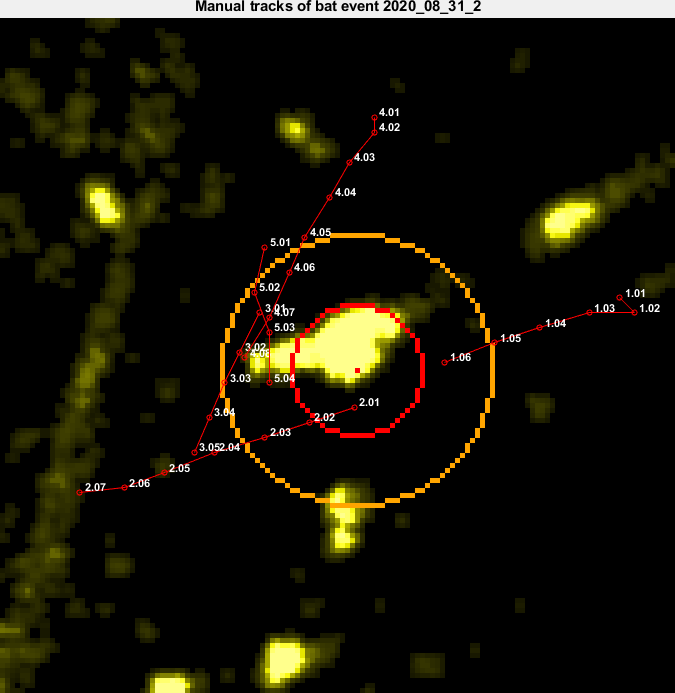

Supplement: S1 File — (ZIP) [file pone.0299153.s003.zip › Manual_tracks_2020_08_31_2.png]

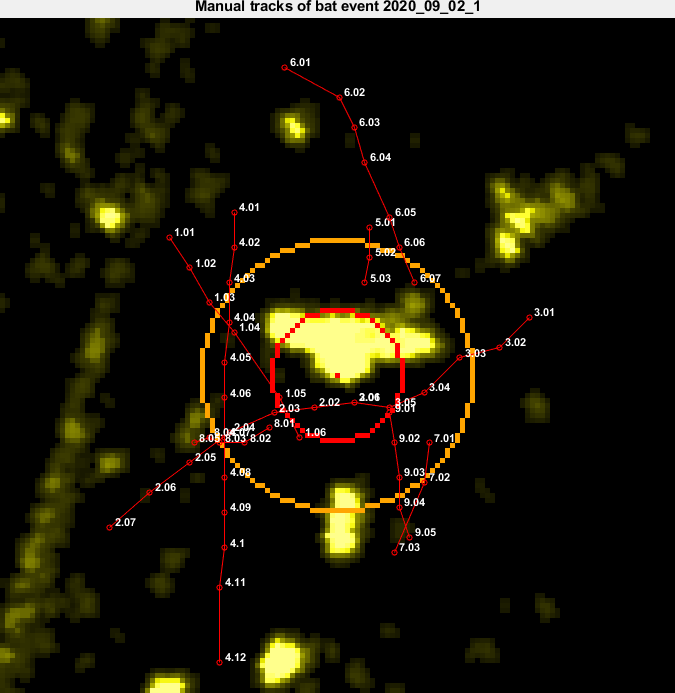

Supplement: S1 File — (ZIP) [file pone.0299153.s003.zip › Manual_tracks_2020_09_02_1.png]

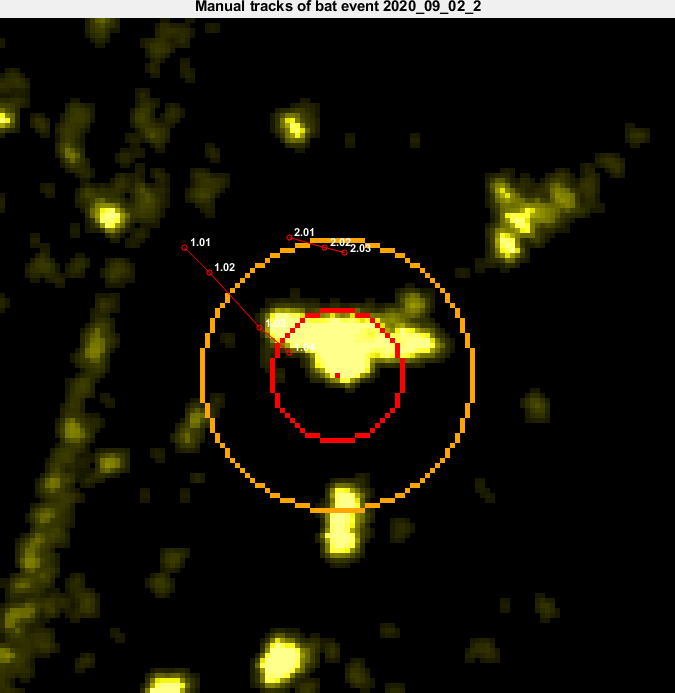

Supplement: S1 File — (ZIP) [file pone.0299153.s003.zip › Manual_tracks_2020_09_02_2.png]

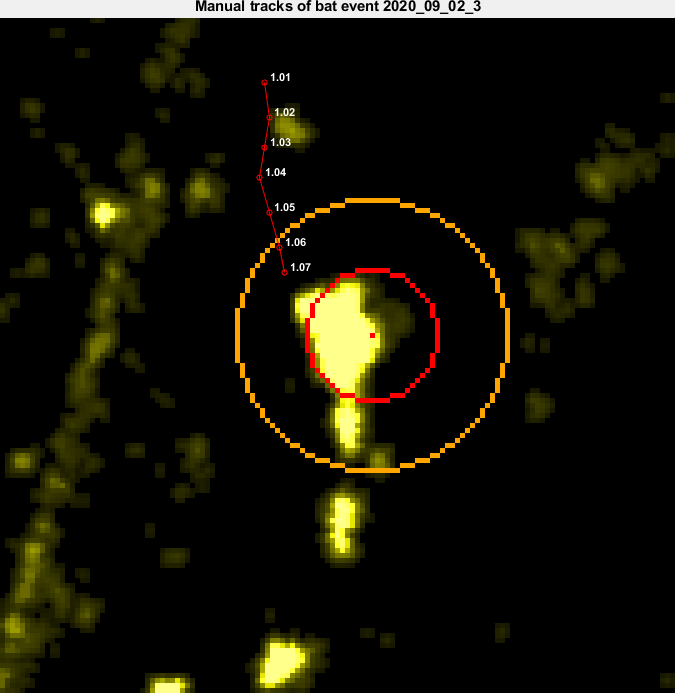

Supplement: S1 File — (ZIP) [file pone.0299153.s003.zip › Manual_tracks_2020_09_02_3.png]

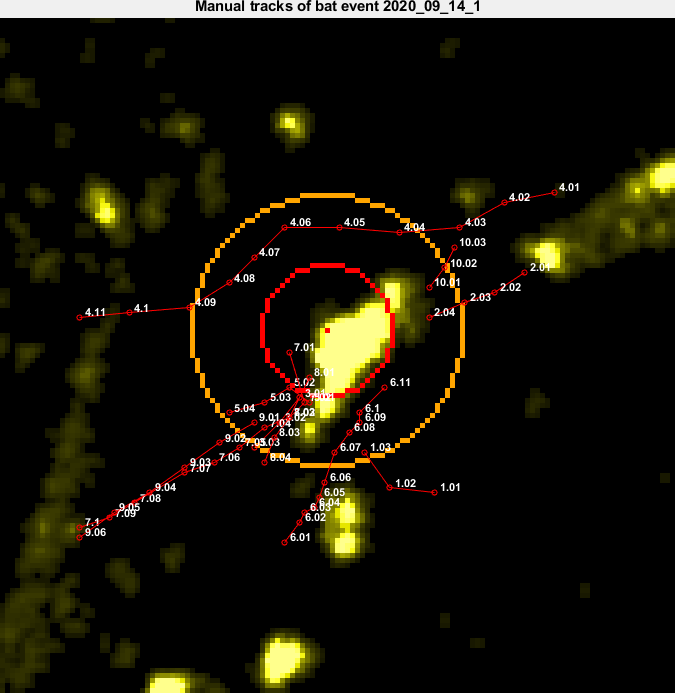

Supplement: S1 File — (ZIP) [file pone.0299153.s003.zip › Manual_tracks_2020_09_14_1.png]

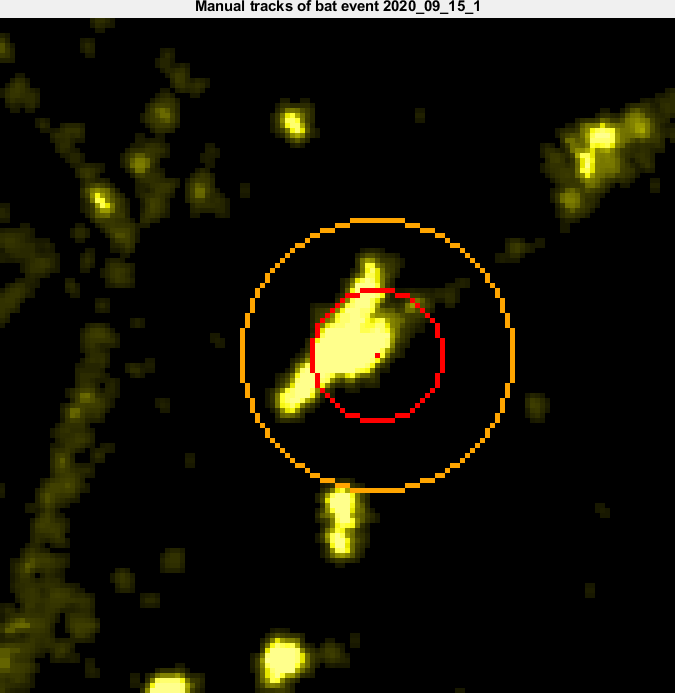

Supplement: S1 File — (ZIP) [file pone.0299153.s003.zip › Manual_tracks_2020_09_15_1.png]

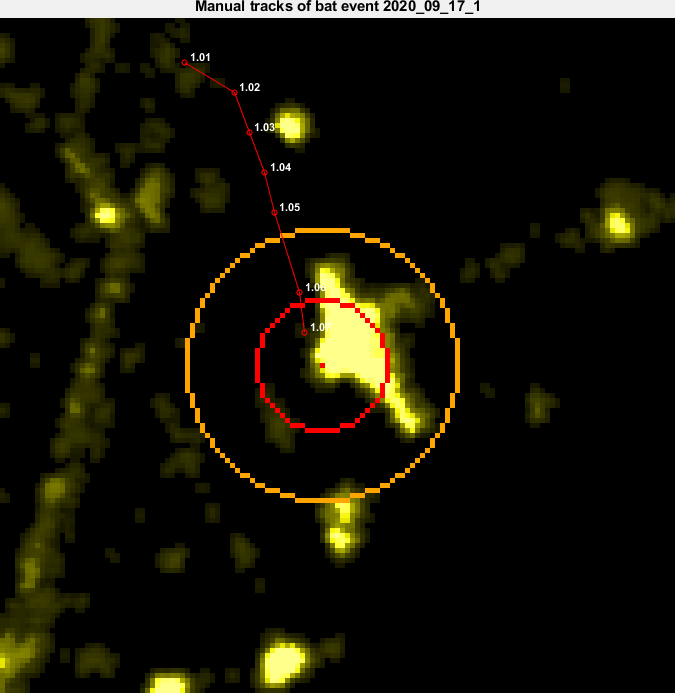

Supplement: S1 File — (ZIP) [file pone.0299153.s003.zip › Manual_tracks_2020_09_17_1.png]

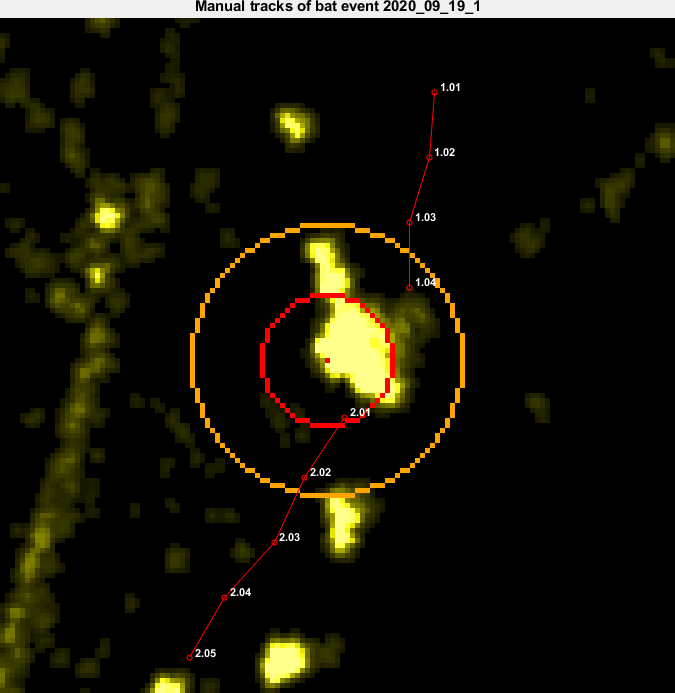

Supplement: S1 File — (ZIP) [file pone.0299153.s003.zip › Manual_tracks_2020_09_19_1.png]

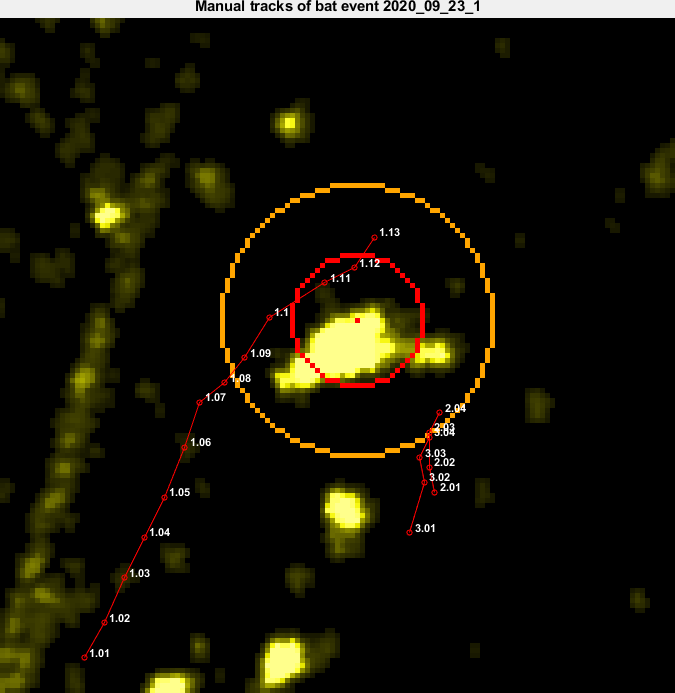

Supplement: S1 File — (ZIP) [file pone.0299153.s003.zip › Manual_tracks_2020_09_23_1.png]

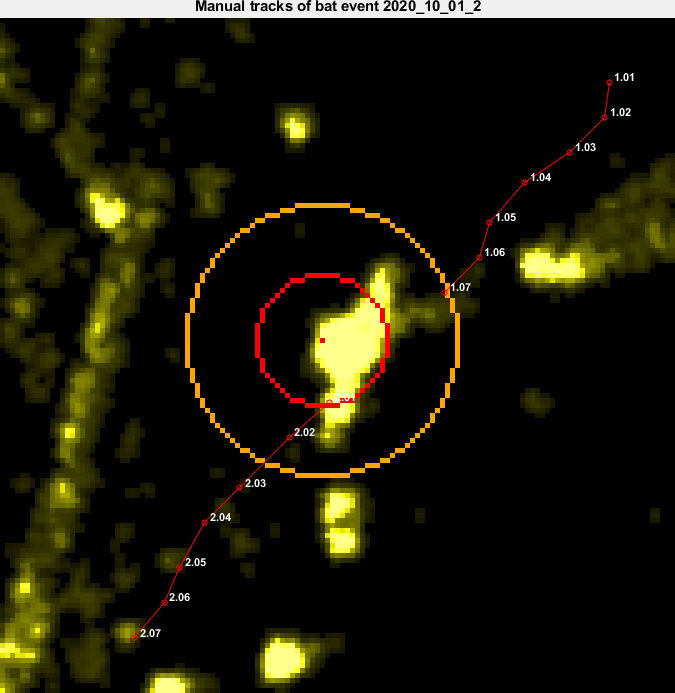

Supplement: S1 File — (ZIP) [file pone.0299153.s003.zip › Manual_tracks_2020_10_01_2.png]
